# Pattern Recognition Techniques Applied to the Study of Leishmanial Glyceraldehyde-3-Phosphate Dehydrogenase Inhibition

**DOI:** 10.3390/ijms15023186

**Published:** 2014-02-21

**Authors:** Norka B. H. Lozano, Rafael F. Oliveira, Karen C. Weber, Kathia M. Honorio, Rafael V. C. Guido, Adriano D. Andricopulo, Alexsandro G. de Sousa, Albérico B. F. da Silva

**Affiliations:** 1 Instituto de Química de São Carlos, USP, São Carlos (SP), 13566-590, Brazil; E-Mail: nbhl_@hotmail.com; 2 Universidade Federal da Paraíba, João Pessoa (PB), 58051-900, Brazil; E-Mails: rfarias.quimica@gmail.com (R.F.O.); kac.weber@gmail.com (K.C.W.); 3 Escola de Artes Ciências e Humanidades, USP, São Paulo (SP), 03828-000, Brazil; E-Mail: kmhonorio@usp.br or kamaho@gmail.com; 4 Universidade Federal do ABC, Santo André (SP), 09210-180, Brazil;; 5 Instituto de Física de São Carlos, USP, São Carlos (SP), 13566-590, Brazil; E-Mails: rvcguido@ifsc.usp.br (R.V.C.G.); aandrico@ifsc.usp.br (A.D.A.); 6 Universidade Estadual do Sudoeste da Bahia, Itapetinga (BA), 45700-000, Brazil; E-Mail: gamasousa@yahoo.com.br

**Keywords:** pattern recognition, adenosine compounds, glyceraldehyde 3-phosphate dehydrogenase, antileishmanial activity

## Abstract

Chemometric pattern recognition techniques were employed in order to obtain Structure-Activity Relationship (SAR) models relating the structures of a series of adenosine compounds to the affinity for glyceraldehyde 3-phosphate dehydrogenase of *Leishmania mexicana* (*Lm*GAPDH). A training set of 49 compounds was used to build the models and the best ones were obtained with one geometrical and four electronic descriptors. Classification models were externally validated by predictions for a test set of 14 compounds not used in the model building process. Results of good quality were obtained, as verified by the correct classifications achieved. Moreover, the results are in good agreement with previous SAR studies on these molecules, to such an extent that we can suggest that these findings may help in further investigations on ligands of *Lm*GAPDH capable of improving treatment of leishmaniasis.

## 1. Introduction

The development of new chemotherapeutic agents against parasitic infections that are effective and have appropriate pharmacokinetic properties is one of the priorities of the World Health Organization (WHO) since it affects the poor and needy around the world [1]. The current situation is illustrated by several factors: a very limited repertoire of drugs, failure to provide safe and effective solutions, and is further aggravated by the constant appearance of new strains of resistant parasites [2–4].

**Figure 1.**
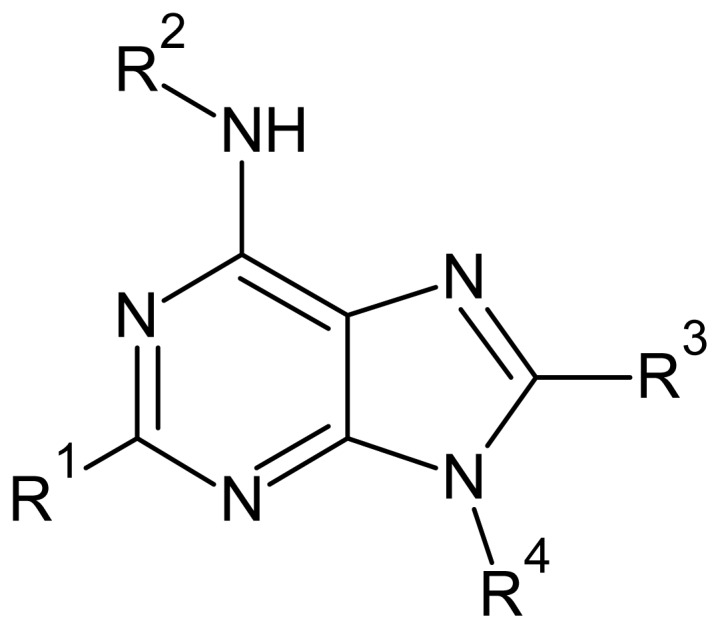
General structure of the adenosine compounds under study.

The glycosomal enzyme glyceraldehyde-3-phosphate dehydrogenase (GAPDH) of the protozoan parasite *Leishmania mexicana*, species of the family Trypanosomatidae, has been identified as a valid target for the design of new drug candidates against one of the most severe parasites distributed through Central and South America [5,6]. *Lm*GAPDH catalyzes the conversion of the substrate 1,3-bisphosphoglycerate (1,3-BPG) in the presence of cofactor NAD+ and inorganic phosphate [7]. The enzyme also plays an important role in controlling the flow of the glycolytic pathway of the parasite [6,7] and, moreover, near the adenosine binding region, many significant structural differences exist between the human GAPDH and *Lm*GAPDH, making it suitable for the design of selective inhibitors [8]. Based on the crystal structures of the NAD, glyceraldehyde-3-phosphate dehydrogenase complexes of humans and *Trypanosoma brucei* (using the adenosine part of the NAD cofactor as a lead structure), anti-parasitic drugs were designed [9–13]. The general structure of these compounds is shown in Figure 1.

In this study, our goal was to build models for the relationships between structures of adenosine derivatives and their affinities to *Lm*GAPDH. In order to identify molecular descriptors that could be related to these affinities, we calculated a large number of electronic, structural and topological descriptors to be used in the multivariate statistical analyses, such as PCA (Principal Component Analysis), HCA (Hierarchical Cluster Analysis), KNN (K-Nearest Neighbor) and SIMCA (Soft Independent Modeling of Class Analogy). Although there are still many challenges in the Quantitative Structure Activity-Relationship (QSAR) field, several methodologies have been developed and employed to model the chemical-biological interactions [14–16]. Therefore, this study sought to find theoretical models able to predict the affinities observed experimentally between inhibitors and the parasite enzyme in order to gather understanding of the important features for such interactions and to plan new chemotherapeutic agents.

## 2. Results and Discussion

### 2.1. Unsupervised Pattern Recognition

The purpose of unsupervised pattern recognition is to find realistic densities or clusters of samples in the space given by the measures, which reflect the possible existence of significant interrelationships. The existence of clusters in the data set is evaluated without using information of the class members [17]. In this category of techniques, we can cite hierarchical cluster analysis (HCA) and principal component analysis (PCA).

#### 2.1.1. Hierarchical Cluster Analysis

In hierarchical cluster analysis (HCA), the distances between pairs of variables/samples are calculated and compared. Small distances between samples imply that they are similar. On the other hand, dissimilar samples will be separated by relatively long distances. HCA starts with each sample defined as its own group, so sample clusters join to form new clusters until all samples are part of one group. The main purpose of HCA is to represent the data in a way that emphasizes natural groupings, in order to allocate and therefore categorize samples. The visualization of the groups corresponding to different classes is achieved in the form of a dendrogram where the class can be easily identified. Different dendrograms can be obtained according to the techniques used to link similar clusters [17].

As mentioned before, after reducing the number of descriptors by calculating W_1–2_, different combinations of them were tested and the best HCA and PCA models were obtained using the following variables: E_LUMO_, QR^2^, QR^4^, molecular volume and polarizability (the calculated values are displayed in the Supplementary Information, Table S1). The types and definitions of these descriptors are listed in Table 1.

**Figure 2.**
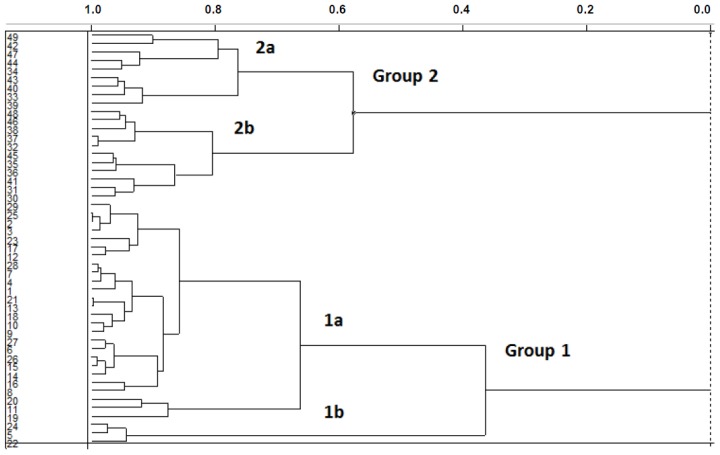
Dendrogram for the training set compounds obtained with incremental linkage.

Figure 2 shows the dendrogram of the samples obtained with the incremental linkage. The branches on the left of the dendrogram represent single samples. The length of the branches that link two groups is related to their similarity; the shorter the distance between branches, the greater the similarity between them. The similarity is plotted along the top of Figure 2, with 1.0 corresponding to an exact duplicate sample and 0.0 indicating the maximum distance and dissimilarity [17].

In Figure 2, the training set compounds appear clustered in two groups: Group 1, containing compounds characterized by higher affinity to *Lm*GAPDH (compounds **1** to **29**), and Group 2, comprising the compounds with lower affinity to *Lm*GAPDH (**30** to **49**). Group 2 is divided into two subgroups: subgroup 2a, in which the substituents (R^1^, R^2^, R^3^ and R^4^) in the basic skeleton are CH_3_, thien-2-yl, CH_3_(C_6_H_4_), H, CH, or CH_2_ (see structures in Table 1) and subgroup 2b, in which most of the compounds have substituents H, Br or CH as R^1^, R^2^, R^3^ and R^4^ groups, indicating that these subgroups are very similar, differing only in the size of some substituents. Another common feature in these groups can be observed as follows: in all compounds of Group 1, the naphthalene moiety appears with and without the substituents hydroxyl, methyl, and methoxy; also, the phenyl moiety appears with and without the substituent methyl as substituents of R^2^. Otherwise, in Group 2 most compounds have hydrogen or other small groups as substituents at R^2^. Additionally, most of the compounds present the naphthalene-substituted attached to the adenine ring and the substituent on the ribose ring at the C2′ position. These findings are in agreement with previous Structure-Activity Relationship (SAR) studies [10–12], which indicate that the ortho substitution in R^2^ naphthalene with hydroxy, methoxy, and methyl substituted, as well as the presence of substituents on the ribose ring at the C2′ position is favorable to high affinity to glyderaldehyde-3-phsphate dehydrogenase (GAPDH). Since the compounds are grouped based on the values of pIC_50_, (Group 1, pIC_50_ ≥ 4.00 and Group 2, pIC_50_ < 4.00), in the following analyses it was considered that Class 1 corresponds to Group 1 and Class 2 corresponds to Group 2.

#### 2.1.2. Principal Component Analysis

Principal Component Analysis (PCA) is a mathematical manipulation of the data matrix where the goal is to represent the variation present in many variables using a small number of principal components (PCs). PCA finds linear combinations of the original independent variables that account for the maximum amount of variation. Thus, the samples plotted in the new space formed by the first two or three PCs (located on the axes instead of the original variables) ensure that the representation of variance on data is optimal. It is important to mention that in multivariate data, none of the original variables completely describe the variation in the data set. Meanwhile, the first principal component is calculated so that it describes the variation in the data set more than any original variable. So, the PCA provides the best possible view of the variability of independent variables, showing the natural grouping in the data set, as well as the existence of outlier samples. It may also be possible to associate chemical meaning to the data emerging from the PCA, and use it as a starting point for comparison with unknown samples.

**Figure 3.**
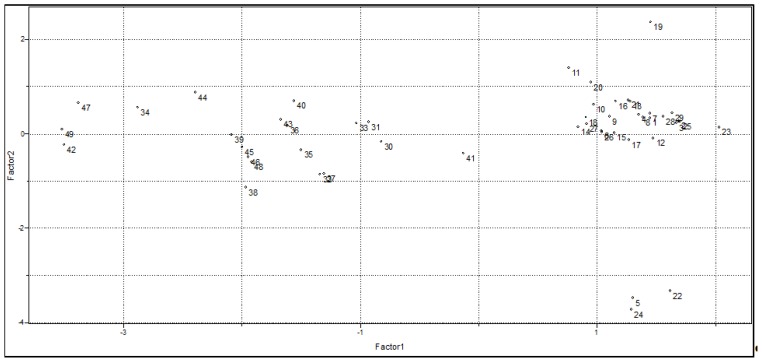
Scores plot of PC1 *vs.* PC2 for the 49 training set compounds.

Figure 3 illustrates the plot of the scores in the first two PCs obtained from combinations of the five variables mentioned above (see Table 1). Together, these components contain 80.3% of the total variance of the original data set provided, being therefore a reliable representation of these first two PCs. In Figure 3 it is possible to notice that PC1 separates the data set in the same two groups observed in the HCA analysis: Group 1 (compounds with higher affinity to GAPDH) and Group 2 (compounds with lower affinity to GAPDH).

Table 2 shows the loadings of each variable on PC1 and PC2. It can be observed that all variables have similar importance to PC1, with the major contribution from QR^2^, Polarizability and Volume, while E_LUMO_ has the highest contribution to PC2.

From Figure 3, we can see that compounds with higher affinity to GAPDH have positive values of PC1 and the variables that have a high positive contribution for PC1 (Table 2) are volume, polarizability and QR^2^. This indicates that steric and electronic effects are very important in understanding the biological activity of the studied compounds. So, to design new *Lm*GAPDH inhibitors with improved biological data, these molecules must have high values of E_LUMO_, QR^2^, volume and polarizability, as well as a low (negative) value of QR^4^. These findings are in good agreement with experimental evidence [19], such as (1) the absence of bulky groups in the sterically favorable region along with sterically unfavorable substituents in the adenine ring can explain the poor activity presented by some compounds; (2) favorable regions for electropositive groups in the general structure of the studied compounds that can perform important interactions in the binding site, for example, interactions with Met39, enhance the biological activity. Besides, favorable regions for negatively charged groups were also observed [19].

### 2.2. Supervised Pattern Recognition

In supervised pattern recognition methods, samples of the training set are previously marked with a known range (a category/class is defined). The primary aim is to develop a rule, which classifies these samples correctly and then apply the same rule for the classification of unknown samples [17]. In this group of methodologies, we can cite KNN and SIMCA techniques.

#### 2.2.1. KNN Results

In the KNN method, the Euclidian distance separating each pair of samples in the training set is calculated and stored in a table of distances. The class to which the nearest neighbors belong is attributed for any particular sample. Thus, the classification obtained can be used to predict the classes of unknown samples.

The descriptors selected using HCA and PCA techniques were employed in our KNN analysis. Considering up to ten nearest neighbors, all of the training set compounds were correctly classified, which shows that all the selected variables have good discriminant ability. An external validation of the KNN classification was performed for this model. As mentioned before, the criterion used for the separation of the training set into two classes of compounds was also used to allocate the 14 test set compounds in the classes, that is, compounds with pIC_50_ ≥ 4.00 are in Class 1, and compounds with pIC_50_ < 4.00 are in Class 2. There was no error of prediction when the distances to 1, 3, 5, 7, 9 and 10 neighbors were calculated. The eight test set compounds with higher affinity were allocated to Class 1 and the six compounds of lower affinity to *Lm*GAPDH were placed in Class 2. This indicates that a reliable classification rule was obtained.

#### 2.2.2. SIMCA Results

SIMCA develops principal component models for each class of compounds in the training set. When the values of the independent variables of a new sample are projected in the PC space of each class, the new sample is allocated in the class to which it best fits. Then, the classification model built can be used for predictions of unknown samples. Furthermore, SIMCA provides diagnostic tools related to other interesting aspects such as discrimination and modeling power, class distances and detection of outliers. The structure of the variance of each class produces information on the complexity of the categories and can also reveal the phenomenon that differentiates one category from another. An additional attractive feature of SIMCA is its realistic prediction of options compared to KNN, since the latter allocate each sample to exactly one class in the training set (the class of nearest neighbors) while SIMCA is able to identify if the sample does not belong to any class or can be a member of both classes.

**Figure 4.**
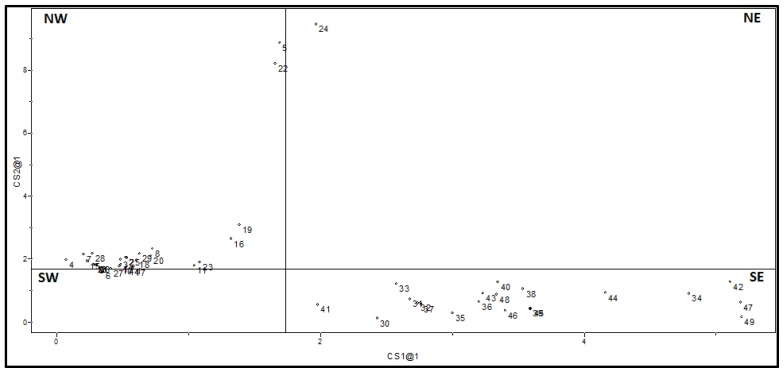
Class distances obtained for the 49 training set compounds.

In this study, the best SIMCA model was built based on the same descriptors used in HCA, PCA, and KNN. Figure 4 shows the distances calculated according to residues of samples when they are adjusted to classes. This plot is divided by two lines that represent the critical residual variances. Compounds that are in the northwest quadrant (NW) belong only to the class of x-axis, because they are at distances small enough to be considered members of this class. Similarly, compounds in the southeast quadrant (SE) are members only of the y-axis class. Compounds in the southwest quadrant (SW) may belong to both classes, while those in the northeast quadrant (NE) do not belong to any class.

In Figure 4 one can see the 20 compounds of lower affinity to *Lm*GAPDH in quadrant SE, which means they are allocated in Class 2 only. On the other hand, the 29 compounds of high affinity to *Lm*GAPDH are in the NW quadrant, belonging to Class 1 only. According to the SIMCA prediction, the compound **24** does not belong to either of the two classes since it is placed in the quadrant NE.

**Figure 5.**
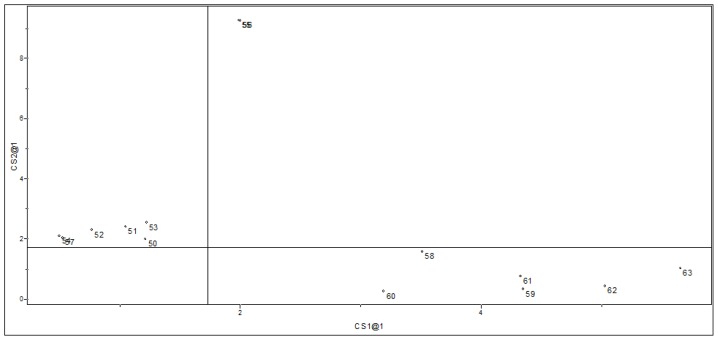
Class distances for SIMCA prediction on the 14 test set compounds.

The SIMCA model obtained from this procedure was subjected to an external validation procedure in order to testify its ability to predict the classes of test set compounds. This validation is shown in Figure 5, which shows the class distances for the test set compounds. It is possible to see that only compounds **55** and **56** were predicted as not belonging to any class, while the other compounds were allocated in their respective correct classes. As with the KNN model presented before, SIMCA also resulted in a predictive model.

Other data trends can be analyzed with the SIMCA classification. The distance between classes (a measure of separation between classes) was calculated as 3.34, showing that the classes are totally different in our model SIMCA (as a rule of thumb, the classes are considered separable when the distance between classes is higher than 3.0 [20]). As with the measures of important variables, the more relevant descriptor for the separation of classes is Volume, which has the highest discriminating power. The descriptor that provides the greatest modeling power is Polarizability, which means that this is the variable that best describes the data set for the two classes observed in the training set. It is interesting to note that the results obtained in this work are in agreement with previous studies which have indicated the importance of steric and electrostatic properties in explaining the affinity of adenosines to *Lm*GAPDH [21,22].

## 3. Experimental Section

### 3.1. Data Set

The adenosine derivatives constituting our data set were extracted from the literature [9–13], with 49 compounds being selected to compose the training set and 14 compounds to constitute the test set (see Table 3). The biological property IC_50_, expressing the concentration of substance required to inhibit 50% of enzyme activity, was converted to pIC_50_ (−log IC_50_) values, which range from 2.22–5.70. The total set of compounds was separated in two classes: compounds with pIC_50_ values higher than or equal to 4.00 were considered as higher affinity compounds (Class 1) and those with pIC_50_ < 4.00 were considered as lower affinity compounds (Class 2).

### 3.2. Geometry Optimization and Descriptor Calculations

The structures of all adenosine derivatives were pre-optimized using the PM3 semi-empirical method [23], and later re-optimized at the Density Functional Theory (DFT) level, using the B3LYP functional [24,25] and the 6-311G** basis set implemented in Gaussian 03 [26]. From these optimized structures, 10 electronic descriptors were calculated, along with eight QSAR descriptors calculated with the HyperChem package [27]. Additionally, 1196 topological descriptors were calculated with Dragon 5.4 [28]. It is important to mention that all calculated descriptors represent the structural, topological and electronic properties of the compounds that can be correlated with the affinities to *Lm*GAPDH experimentally observed.

### 3.3. Variable Selection and Chemometric Analyses

In order to reduce the dimensionality of the descriptor set, the Fisher’s weights (W_1–2_) for each descriptor were calculated. Thus, we were able to select the variables showing good discriminating power to distinguish between high and low affinity compounds. W_1–2_ for the *i*-th descriptor and for samples belonging to classes 1 and 2 is calculated using Equation (1):

(1) $$W_{1-2}(i)=\frac{{\left[{\bar{X}}_{i}(1)-{\bar{X}}_{i}(2)\right]}^{2}}{S_{i}^{2}(1)+S_{i}^{2}(2)}$$

where *X̄_i_* are the mean values of descriptors considering samples from each class (1 and 2) and *S_i_^2^* are the variances for each class.

The 185 descriptors with significant Fisher’s weight (that is, W_1–2_ > 100.0) were selected as those that have the best ability to discriminate between compounds with high and low affinity to *Lm*GAPDH. After this procedure, we tested different combinations until good discriminations in HCA and PCA were found, with no sample being placed in the wrong group.

### 3.4. Pattern Recognition Analyses

All chemometric (pattern recognition) analyses were performed using Pirouette 3.1 [29], after applying the autoscaling preprocessing technique in order to give the same importance to all of the variables/descriptors. The pattern recognition techniques employed in this study can be classified in two categories: unsupervised pattern recognition (HCA and PCA) and supervised pattern recognition (KNN and SIMCA). HCA helped us to define the class to which the compounds belong, while PCA provided an initial knowledge of the basic structure of the data set. KNN and SIMCA, two methods based on the assumption that closer samples are more likely to belong to the same class, were employed to build classification models of affinity to *Lm*GAPDH. Except for HCA, each one of these methods was employed here in two steps. First, a model was built and refined, based on the compounds of the training set, and then it was used to make predictions for unknown samples (compounds in the test set).

## 4. Conclusions

In this study, chemometric pattern recognition approaches were successfully applied, for the first time, in order to obtain predictive SAR models for adenosine compounds. The aim of this study, involving adenosine derivatives, their affinities to *Lm*GAPDH and pattern recognition techniques, is to understand the fundamental effects involved in the interaction between the bioactive ligands and the biological target. The computational procedure employed here has enabled discrimination of the studied compounds, with higher (Class 1) and lower (Class 2) affinities to *Lm*GAPDH, through molecular descriptors obtained by quantum chemical calculations (E_LUMO_, QR^2^, QR^4^, Volume and Polarizability), differently from previous studies where more complex calculations were required [21] or only topological descriptors were able to provide statistically validated QSAR models [22]. All pattern recognition models obtained in the present work have shown internal consistency and were externally validated with a set of test compounds. Furthermore, the characteristics of the compounds studied here, in each group (Class 1 and Class 2), are in agreement with previous empirical SAR/QSAR studies on adenosine derivatives [10–12], in such a way that the pattern recognition models obtained in this work can be deemed helpful in the design of new adenosine compounds that may be able to inhibit *Lm*GAPDH.

## Supplementary Information



## Figures and Tables

**Table 1.** Symbols, types and definitions of selected descriptors [18].

Descriptor	Type	Definition
E_LUMO_	Electronic	Energy of the lowest unoccupied molecular orbital
QR^2^	Electronic	Charge at substituent R^2^
QR^4^	Electronic	Charge at substituent R^4^
Polarizability	Electronic	Molecular polarizability
Volume	Geometrical	Solvent-accessible surface-bounded molecular volume

**Table 2.** Loading values of the selected variables.

Variable	PC1	PC2
ELUMO	0.087	−0.818
QR2	0.497	0.299
QR4	−0.305	0.482
Volume	0.575	0.042
Polarizability	0.566	0.081

**Table 3.** Chemical structures and pIC_50_ values for training and test set compounds.

Training set compounds					
Cpd	Structure	pIC_50_	Cpd	Structure	pIC_50_
**1**	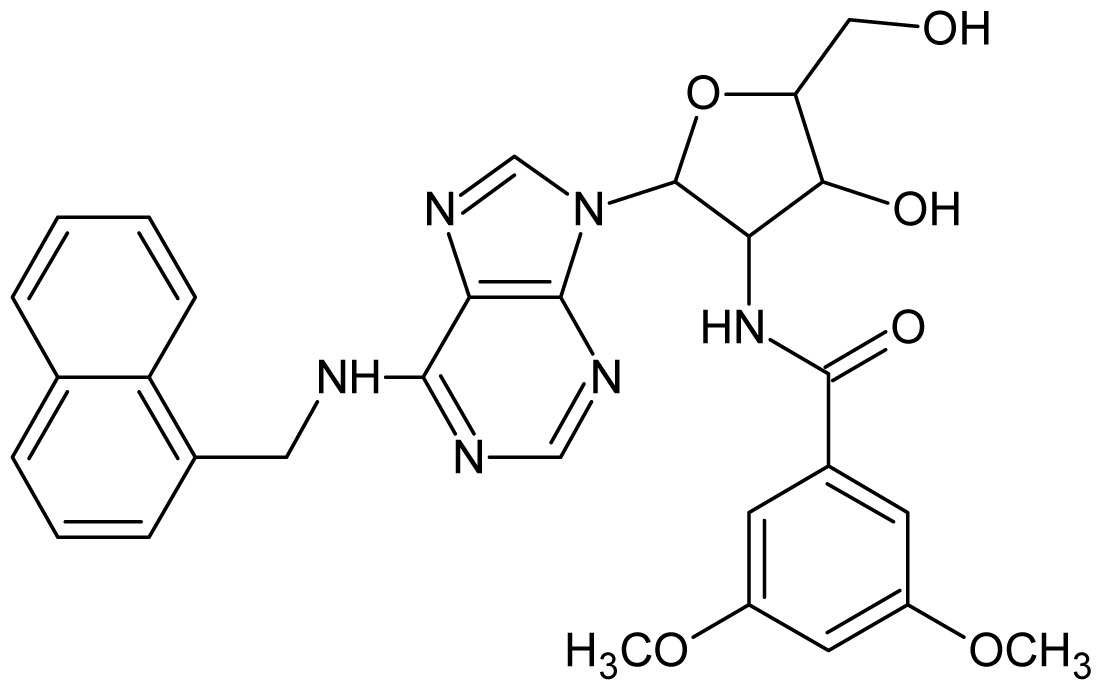	5.70	**2**	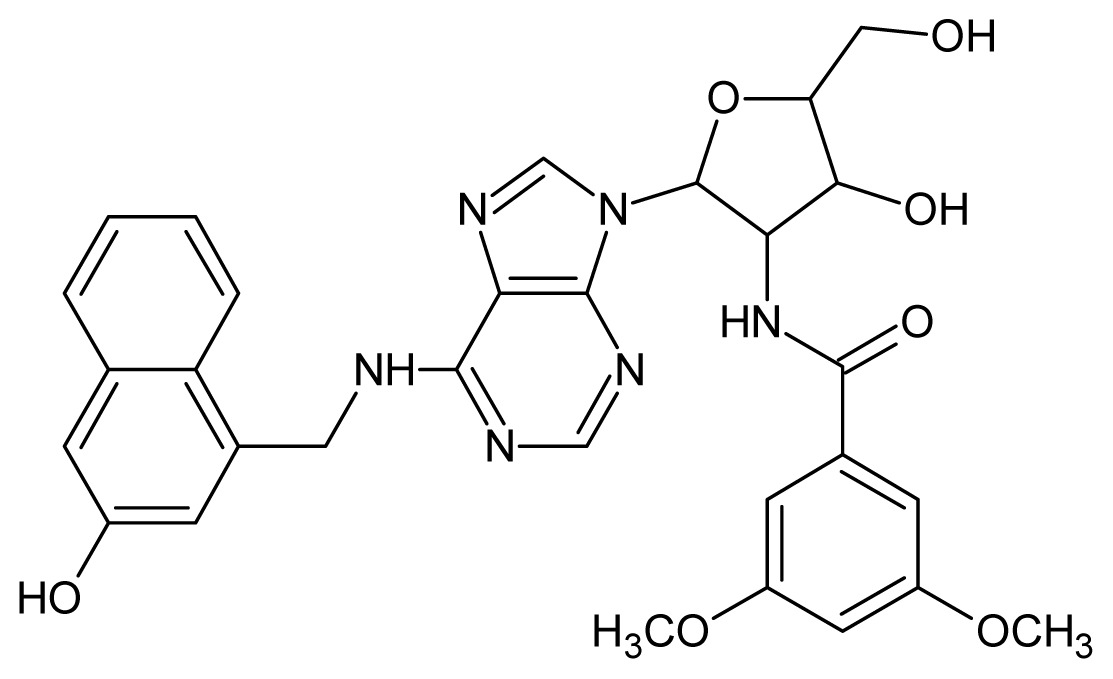	5.70
**3**	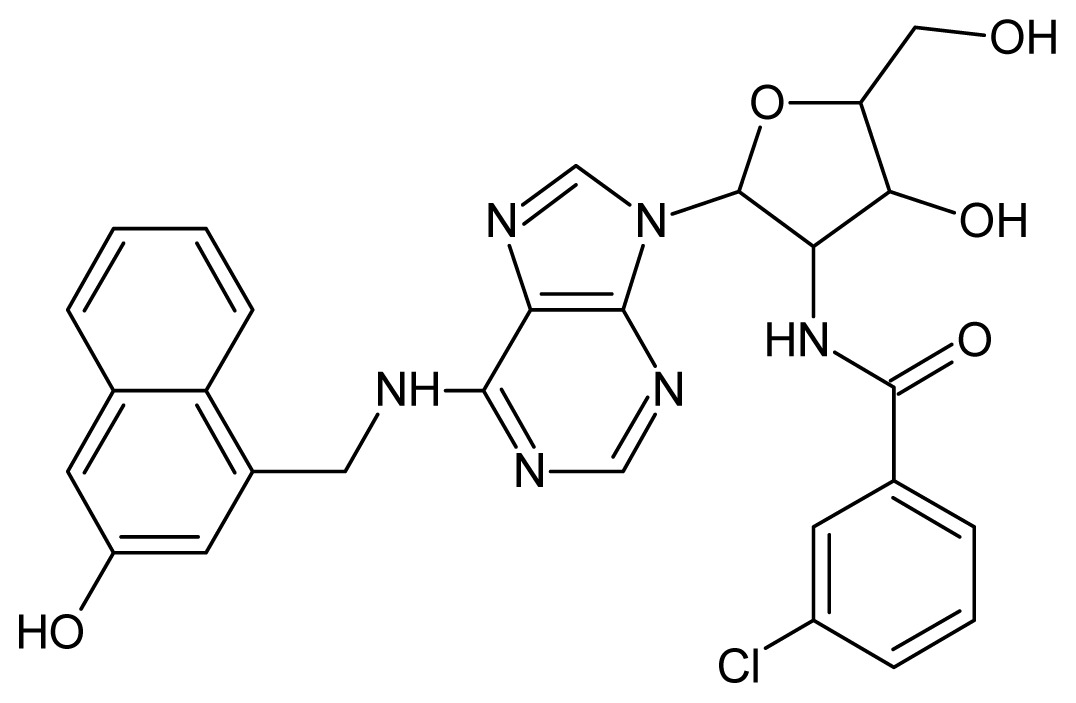	5.70	**4**	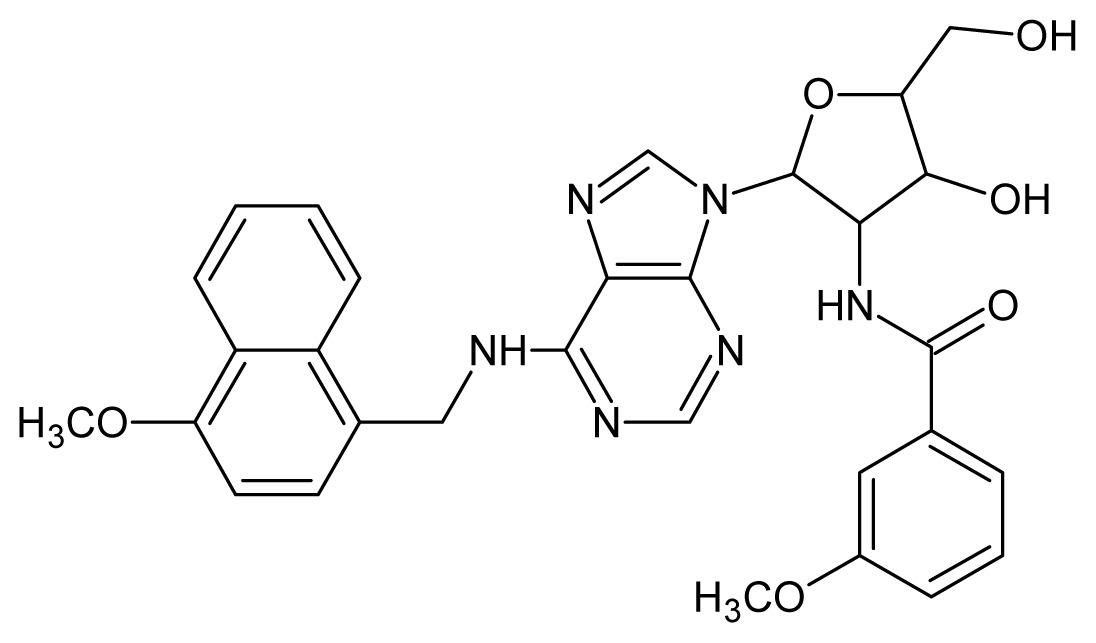	5.70
**5**	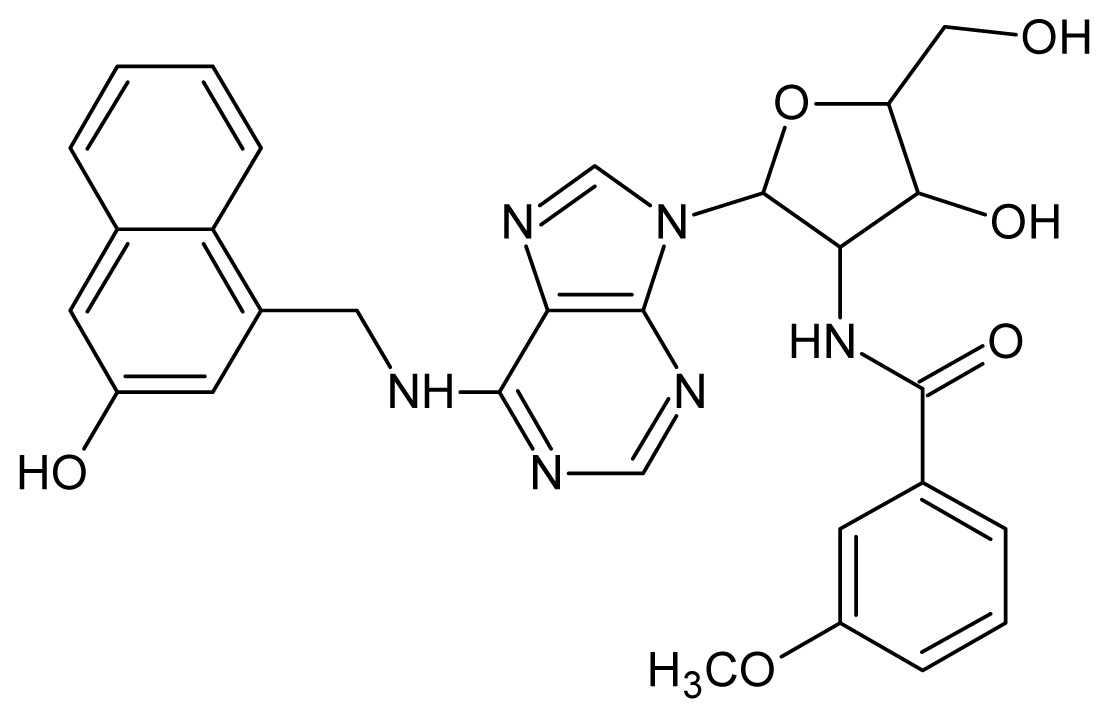	5.40	**6**	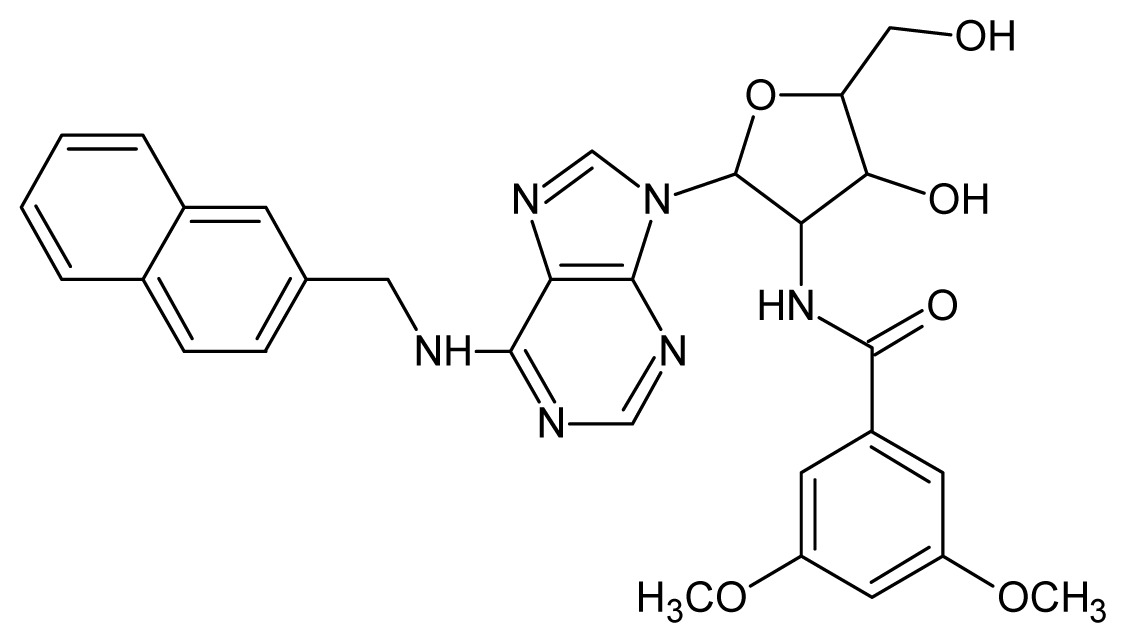	5.30
**7**	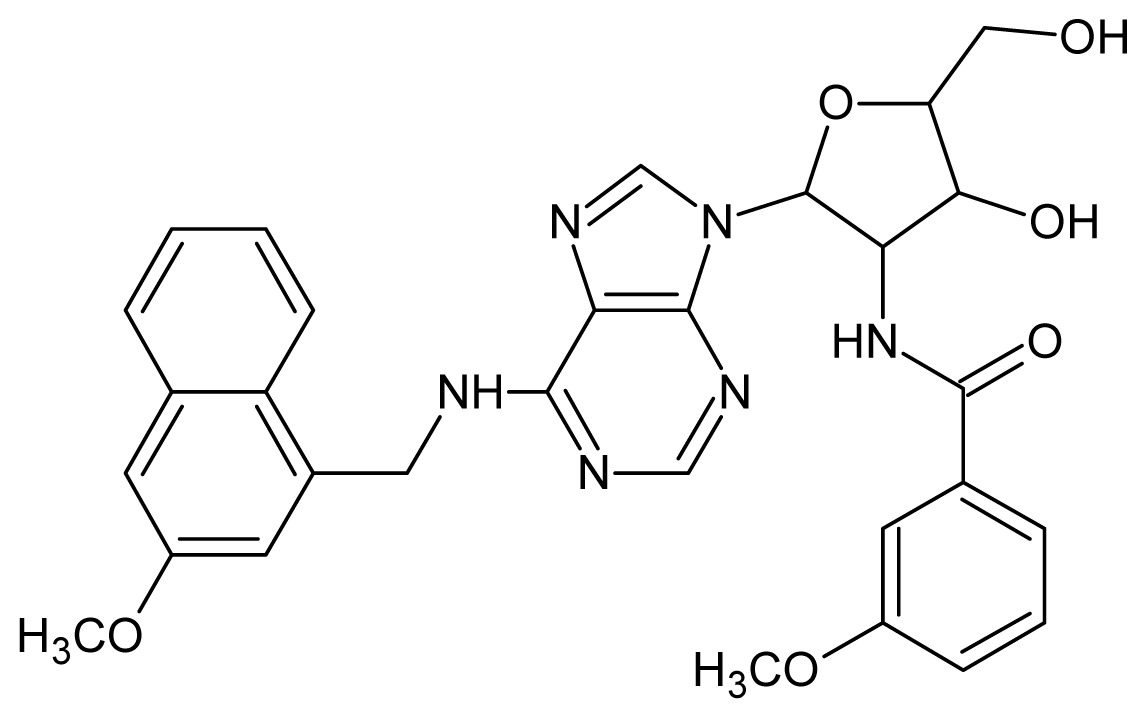	5.30	**8**	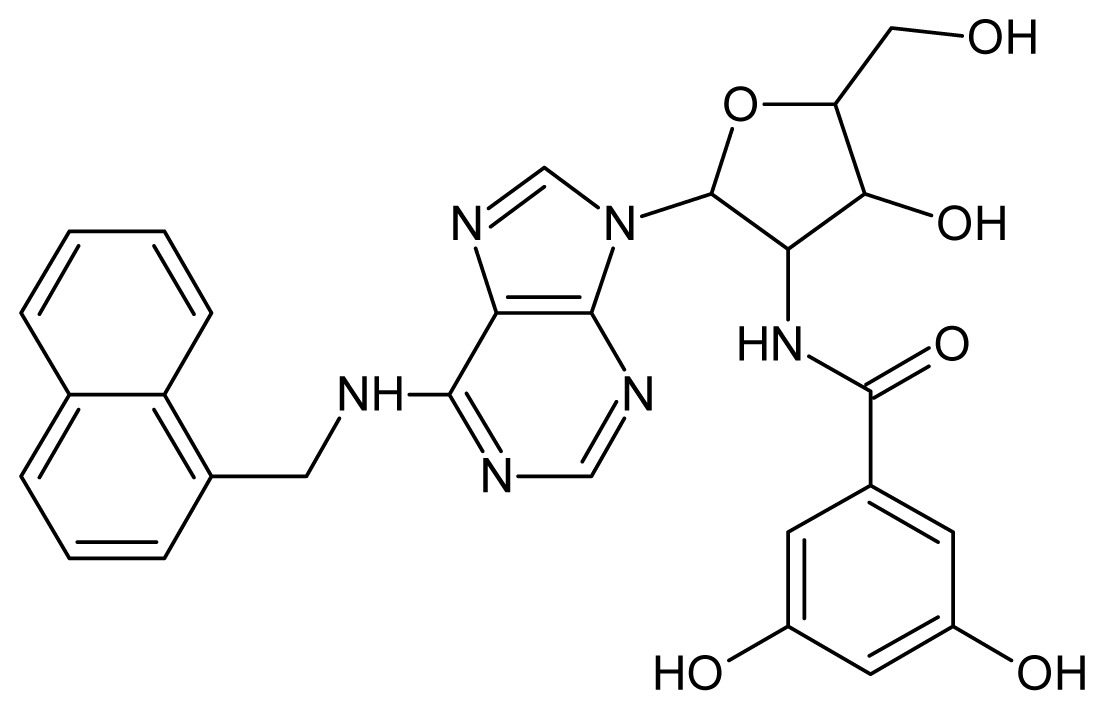	5.26
**9**	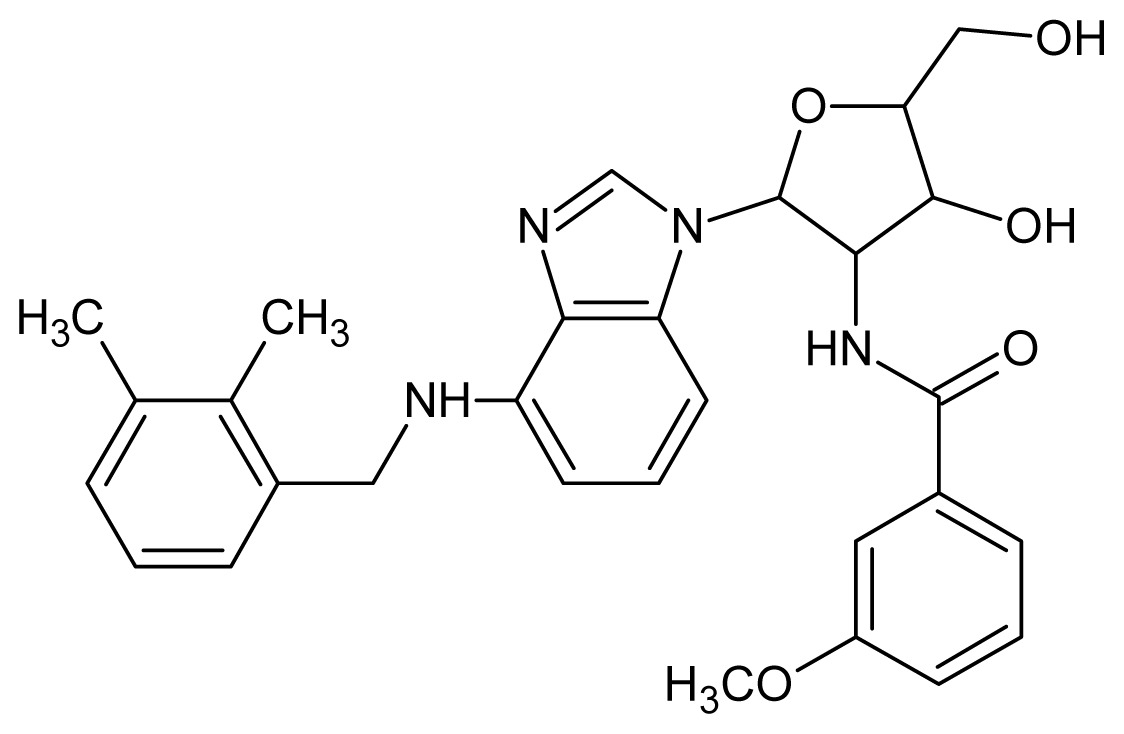	5.22	**10**	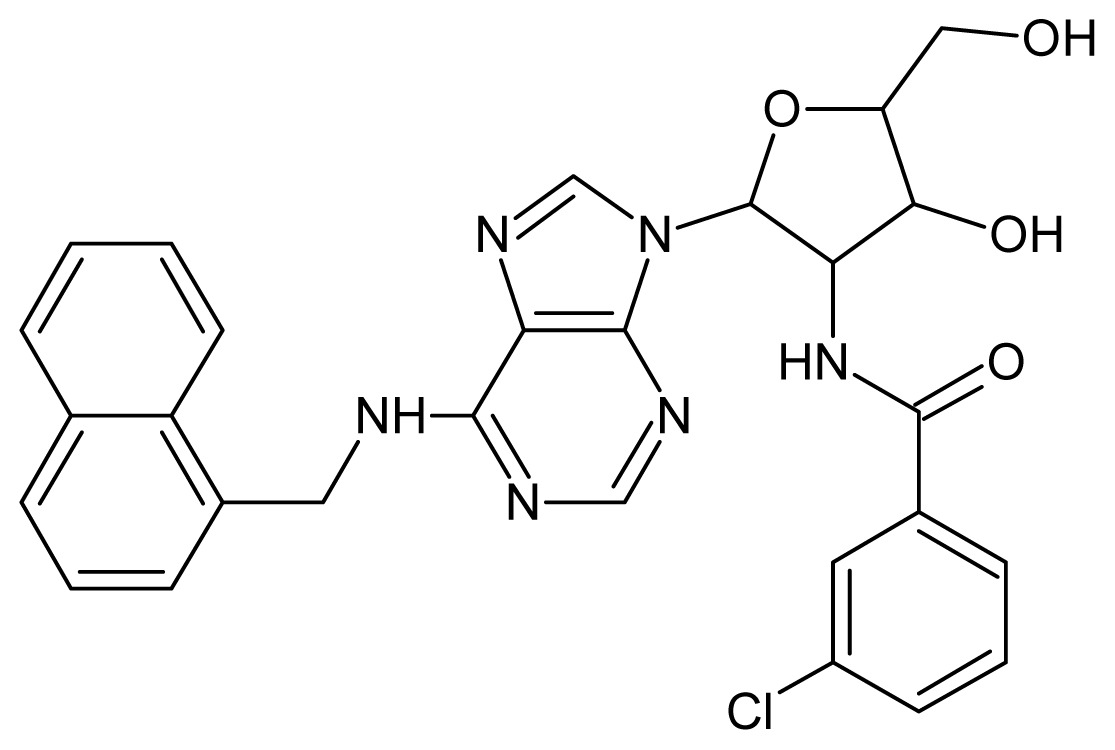	5.00
**11**	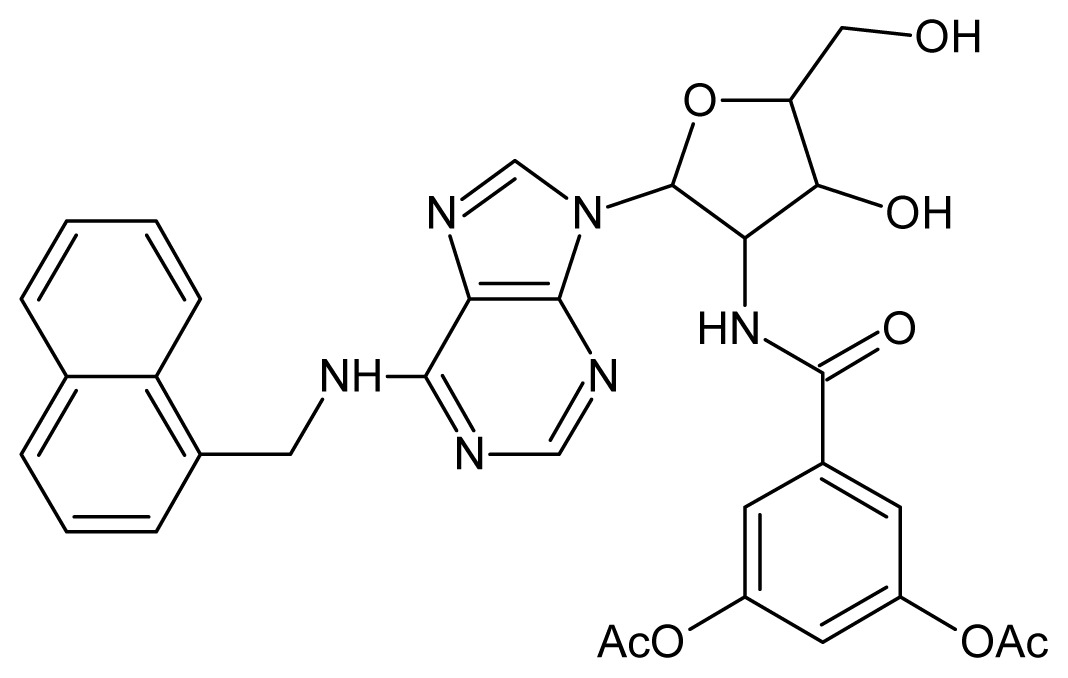	5.00	**12**	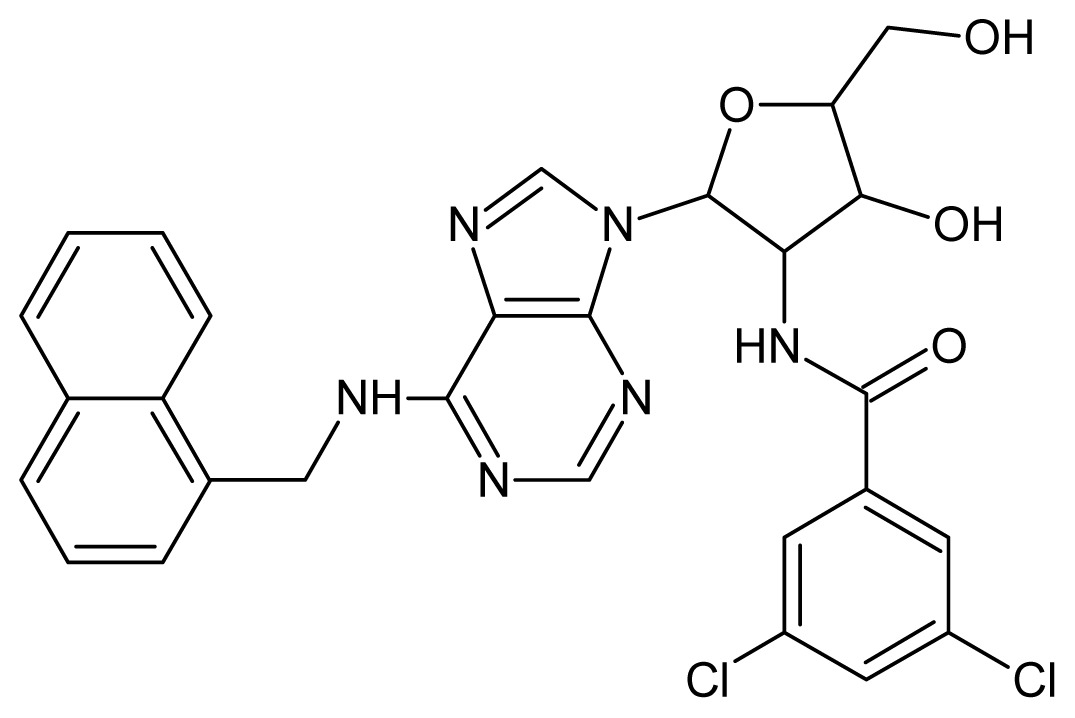	5.00
**13**	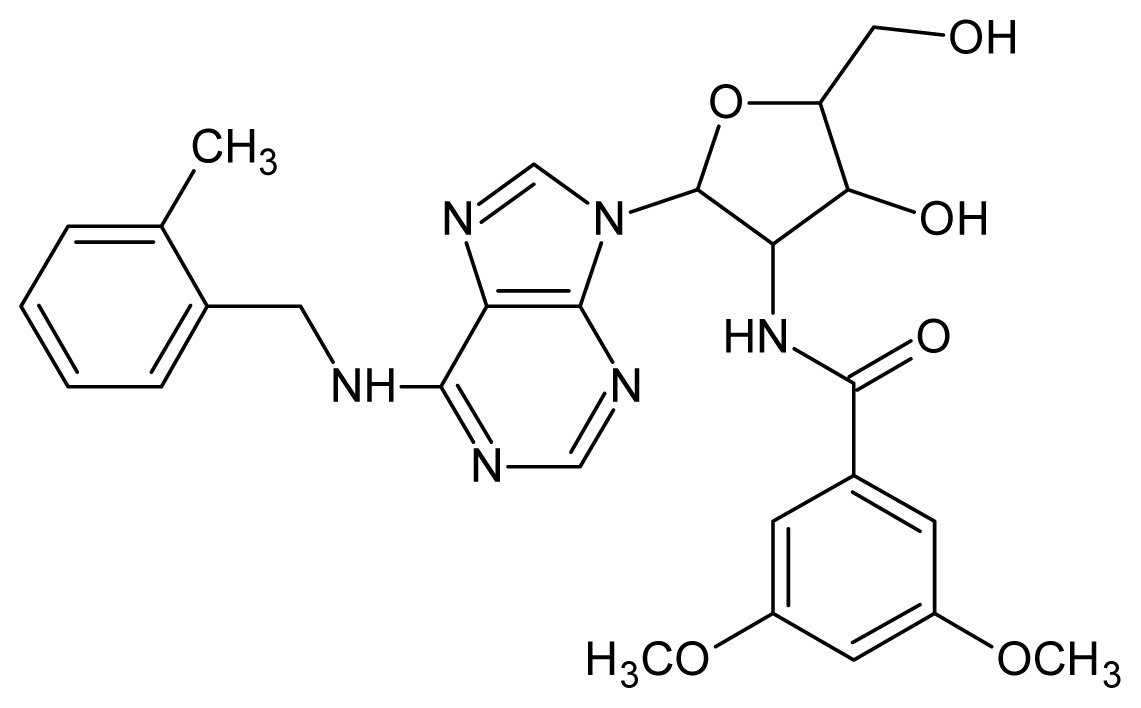	4.92	**14**	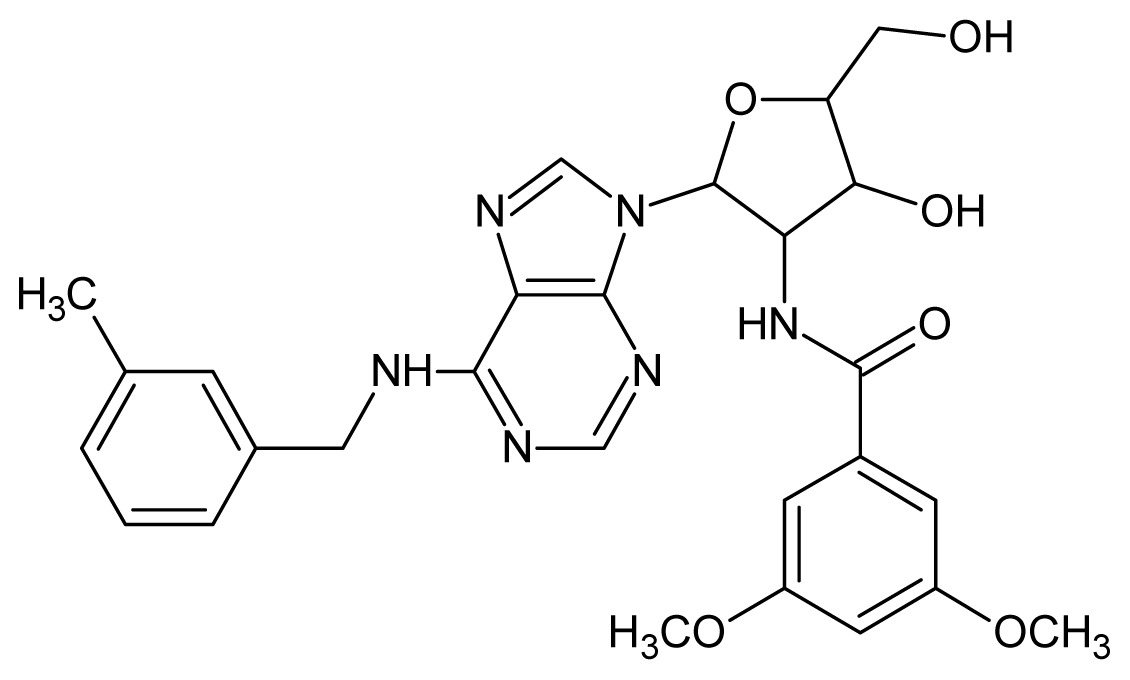	4.70
**15**	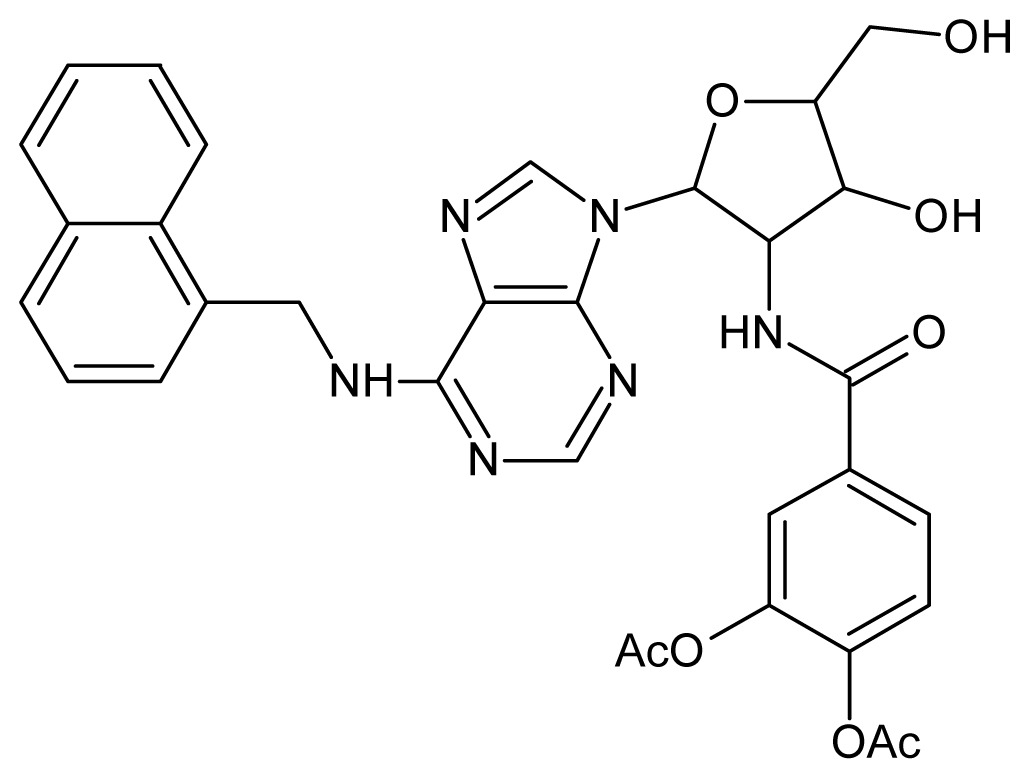	4.60	**16**	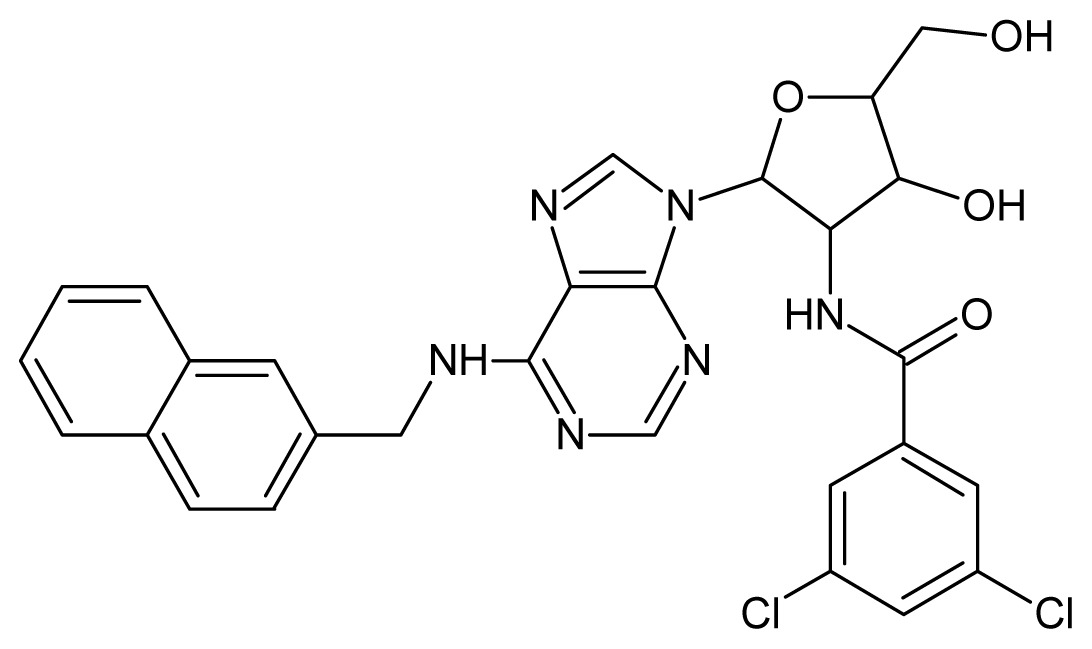	4.60
**17**	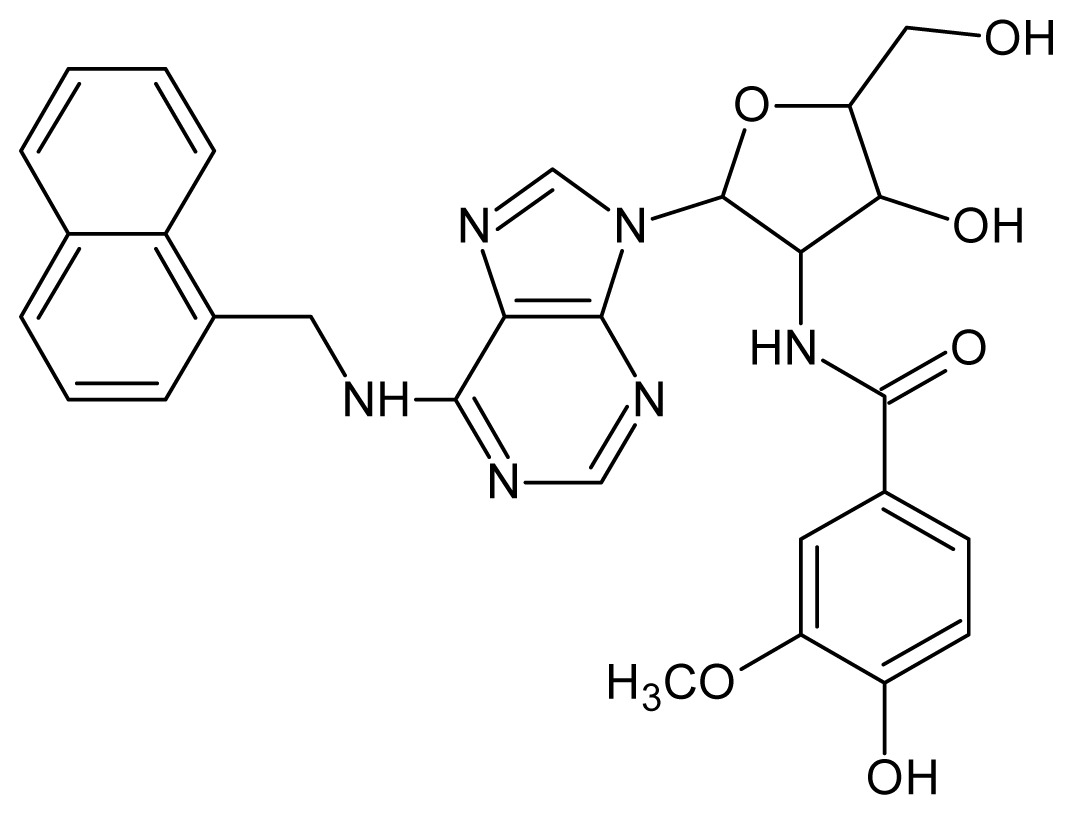	4.60	**18**	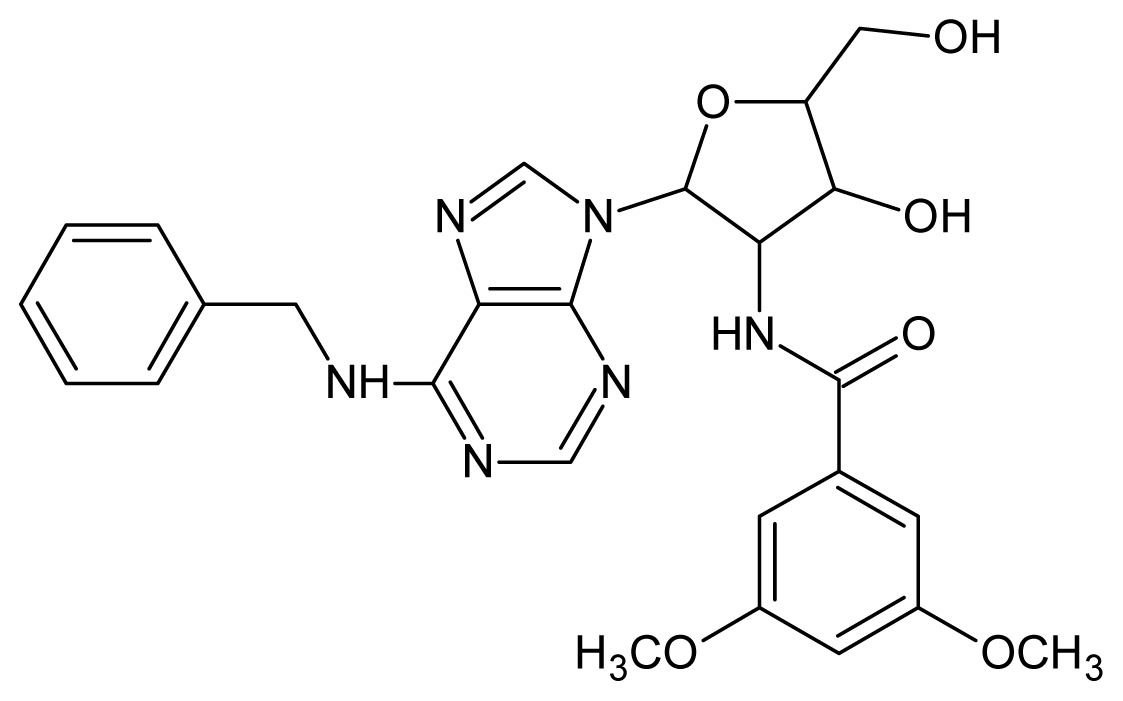	4.60
**19**	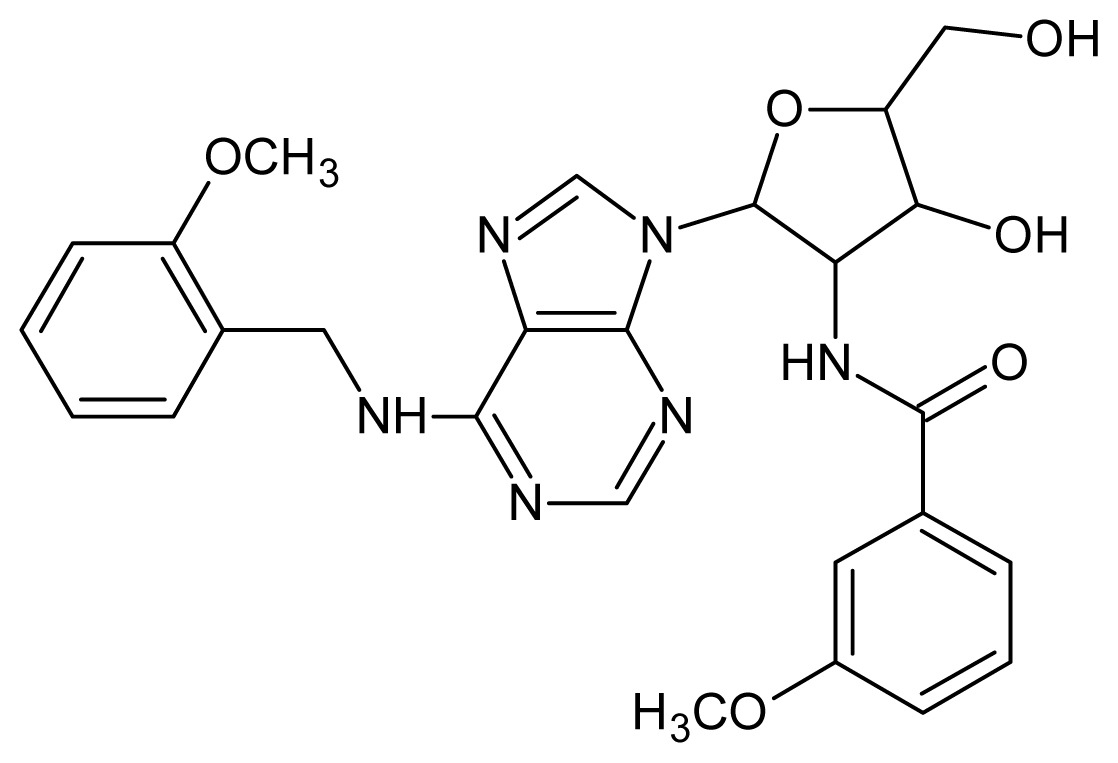	4.60	**20**	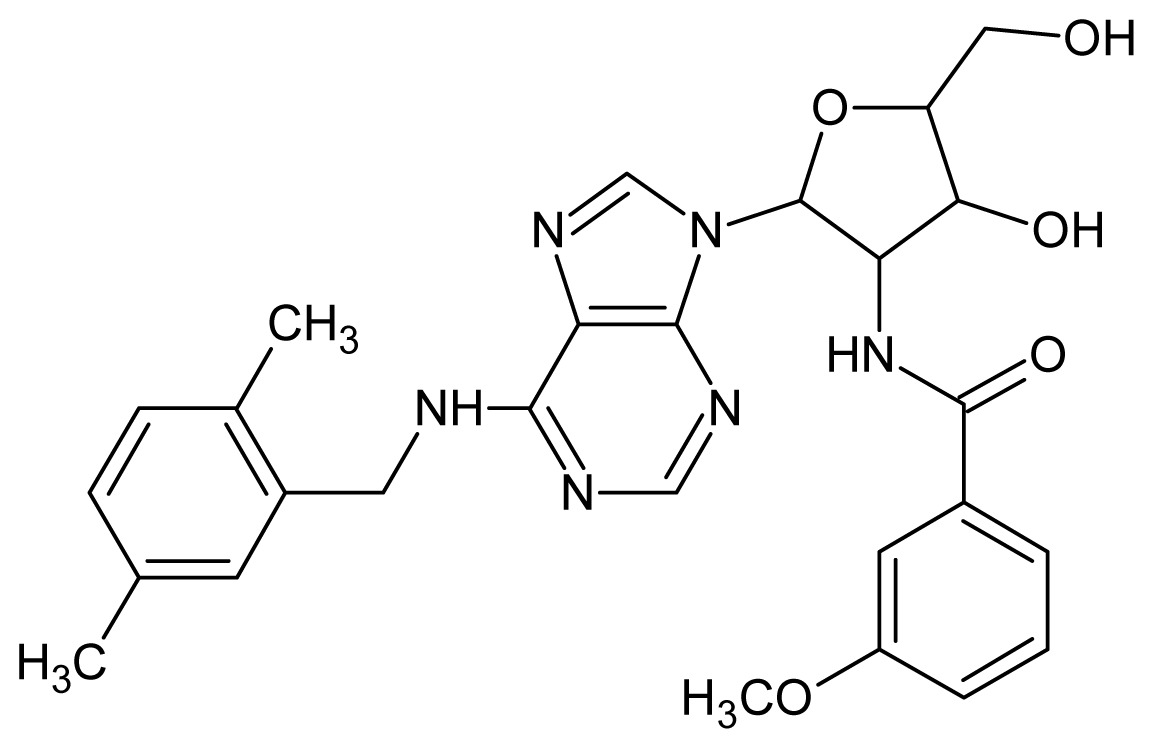	4.60
**21**	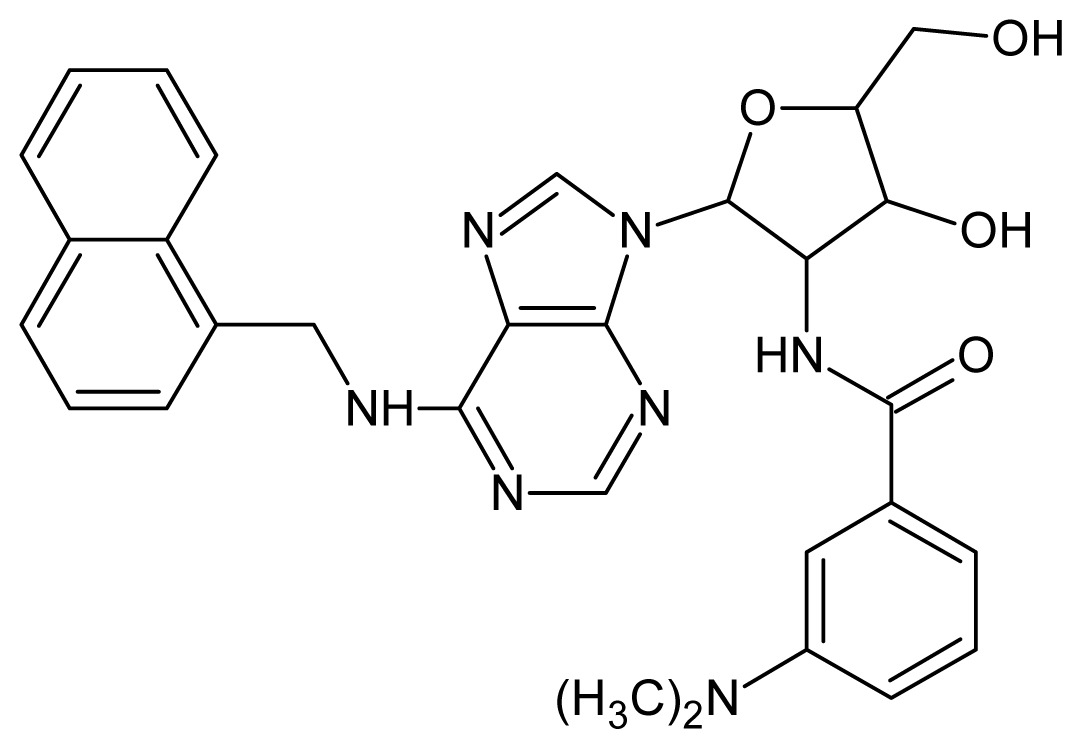	4.60	**22**	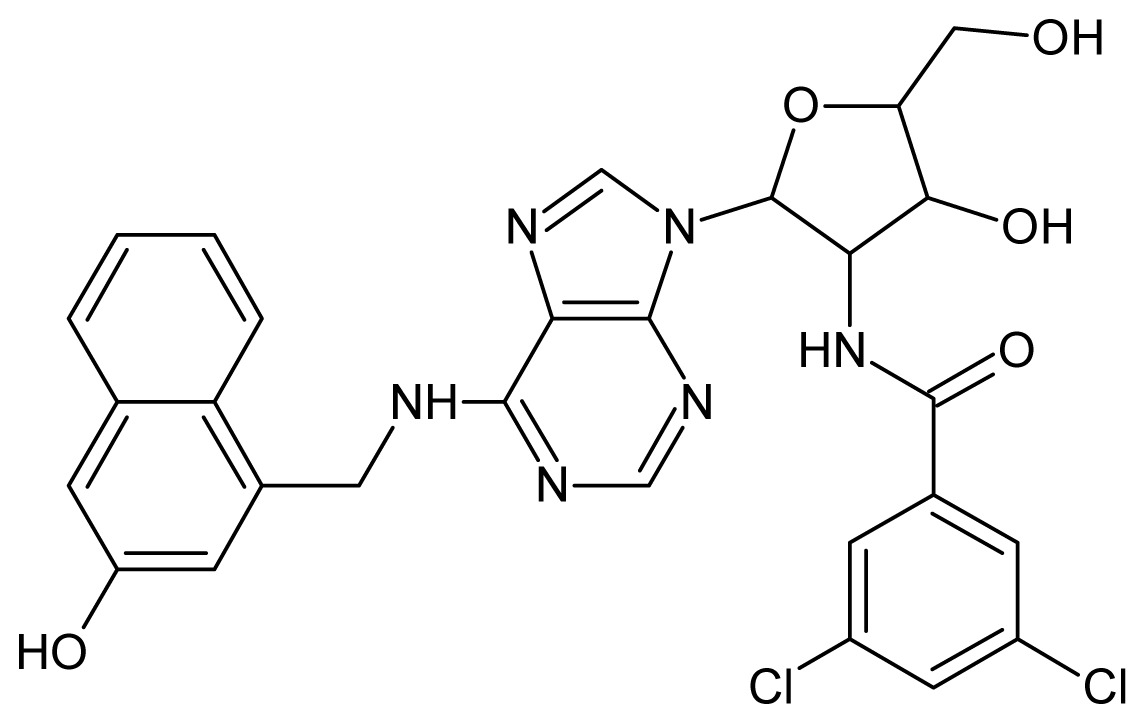	4.60
**23**	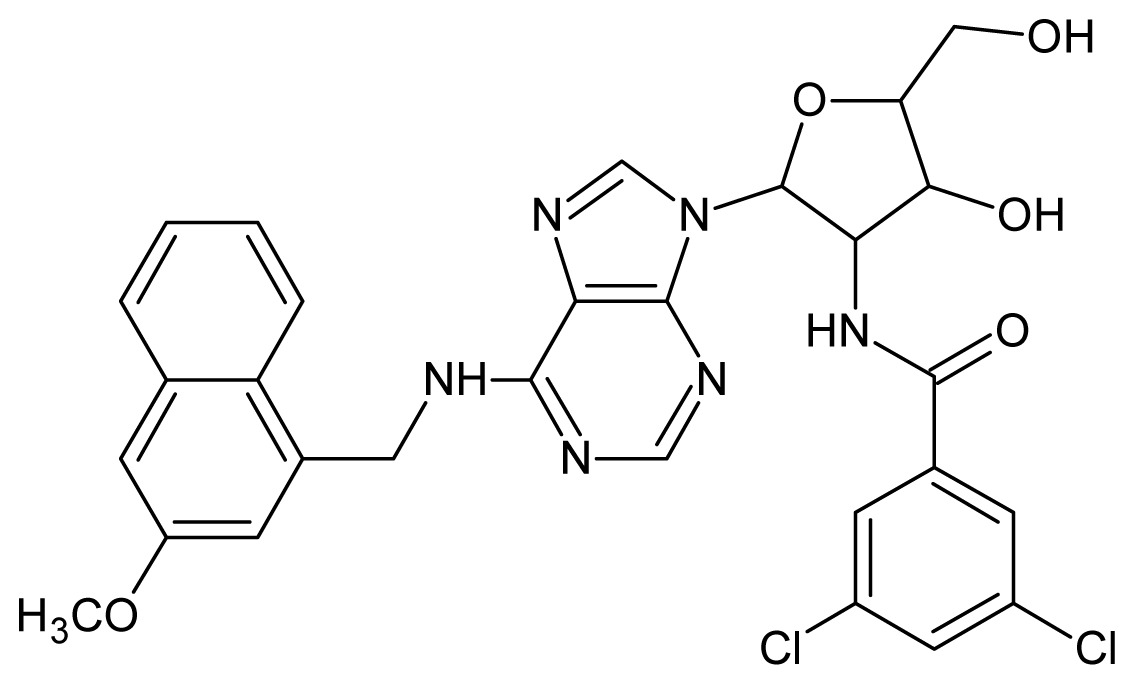	4.60	**24**	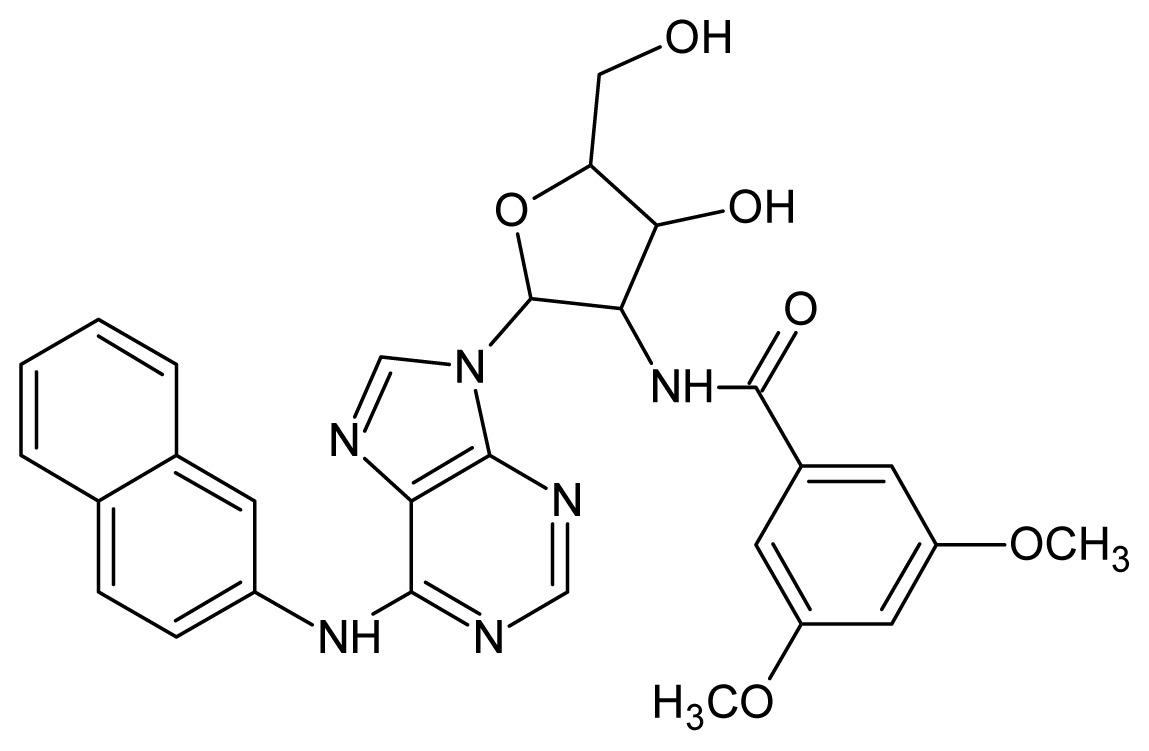	4.43
**25**	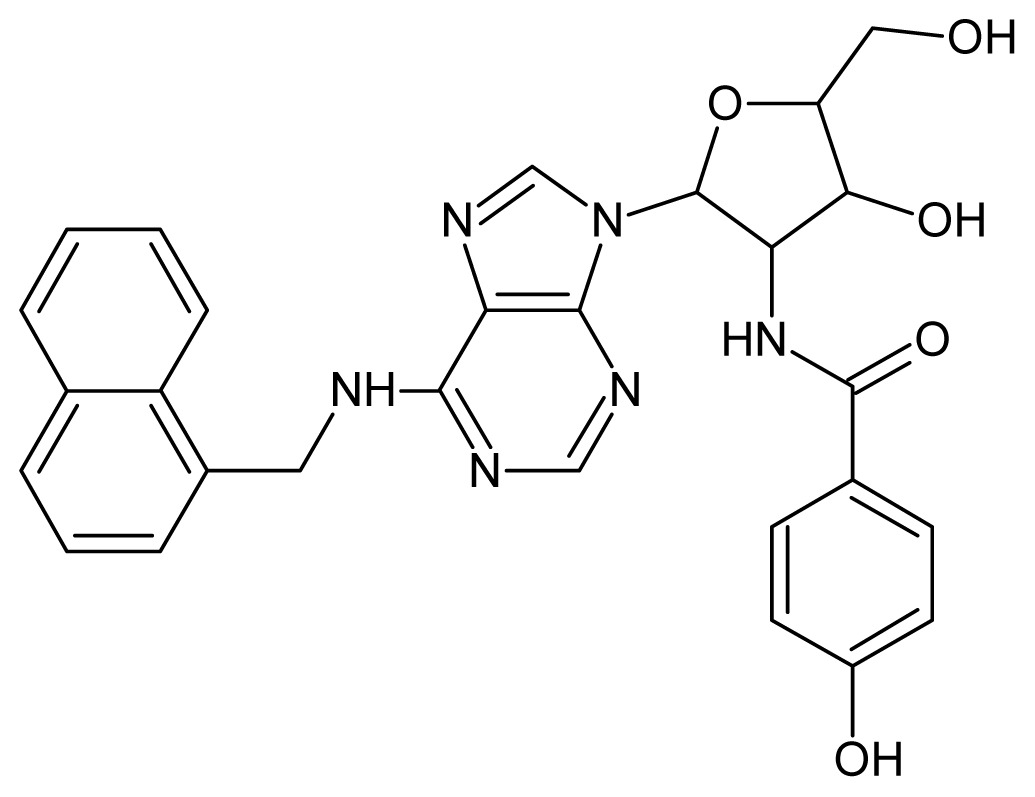	4.22	**26**	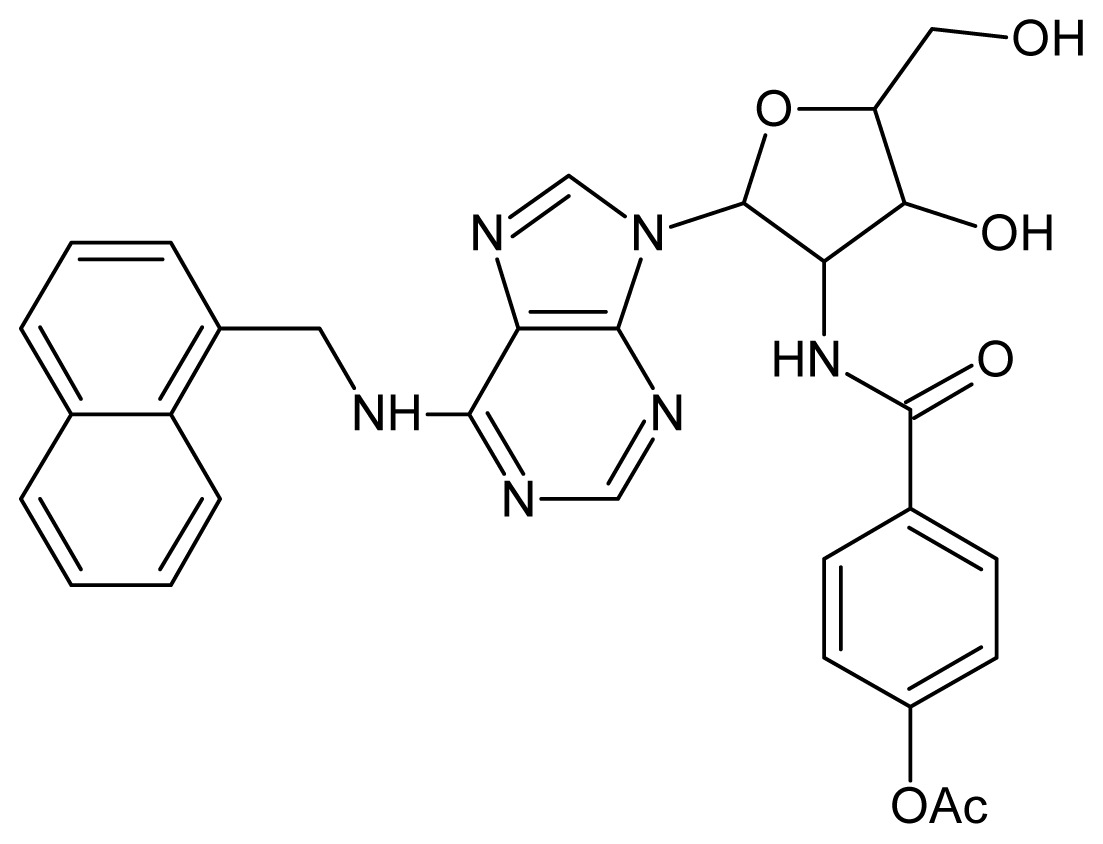	4.10
**27**	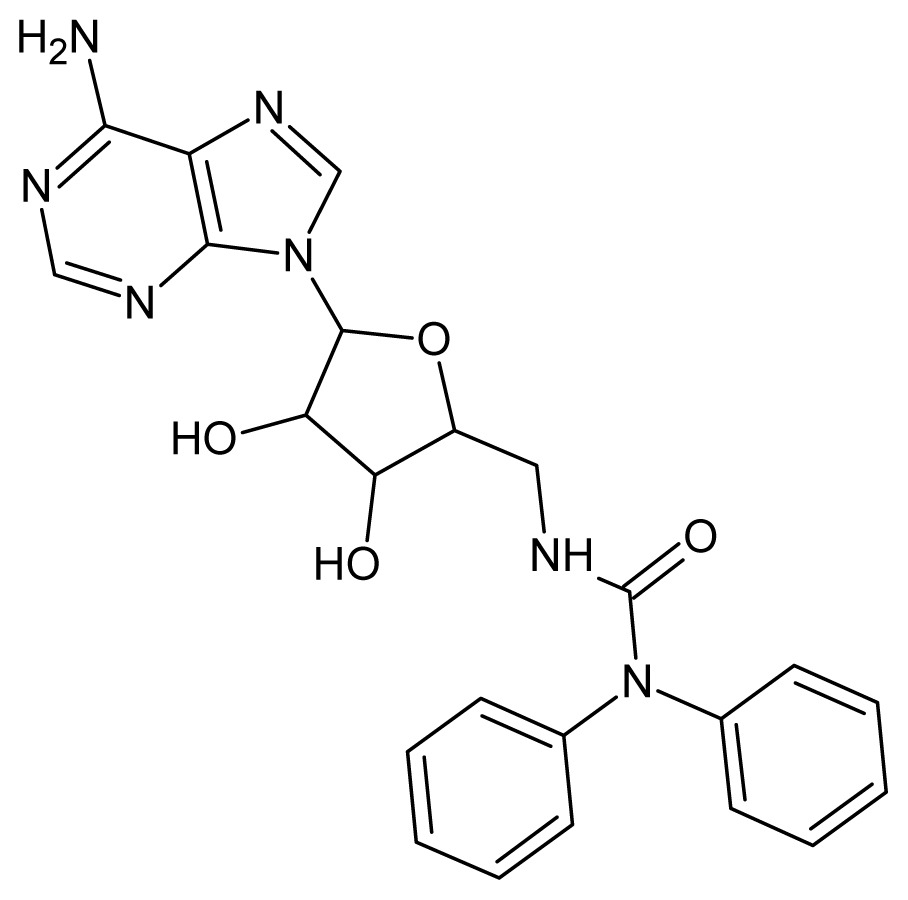	4.10	**28**	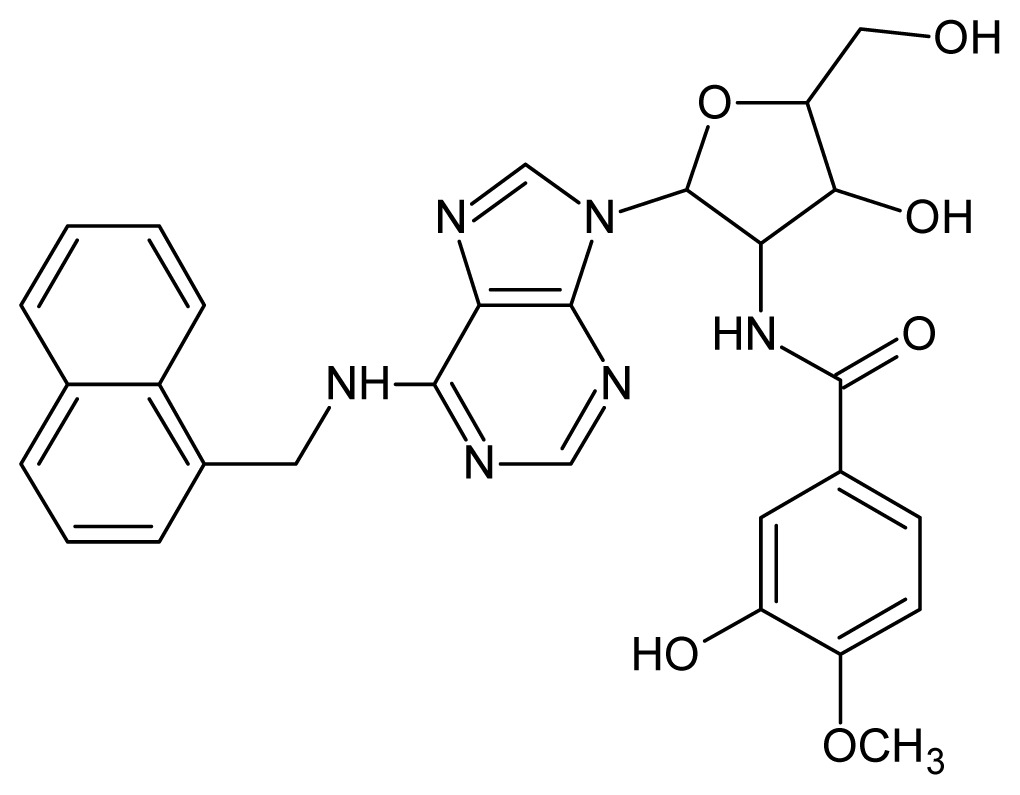	4.08
**29**	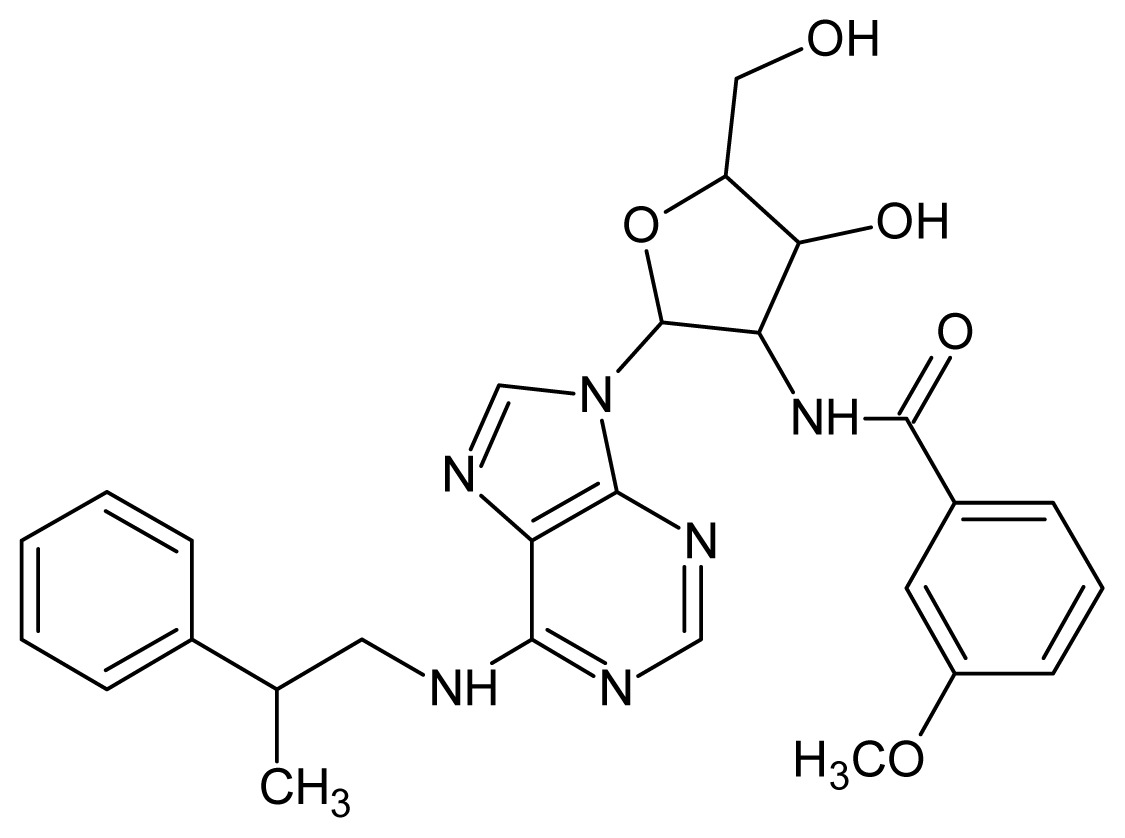	4.00	**30**	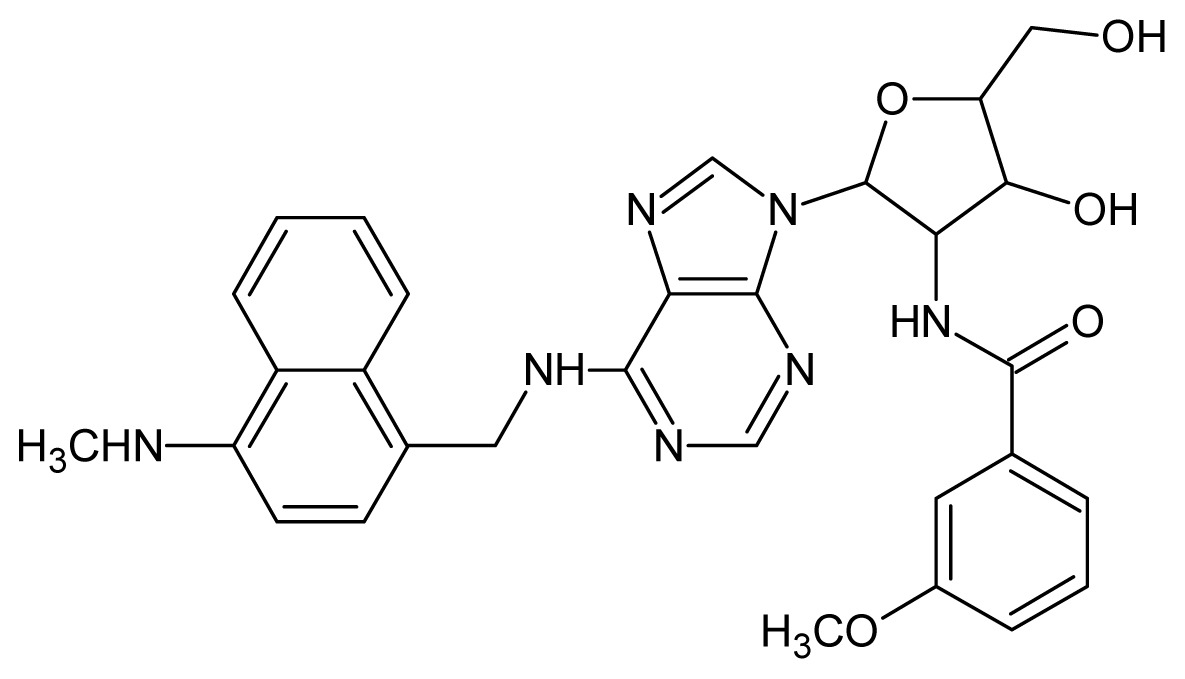	3.70
**31**	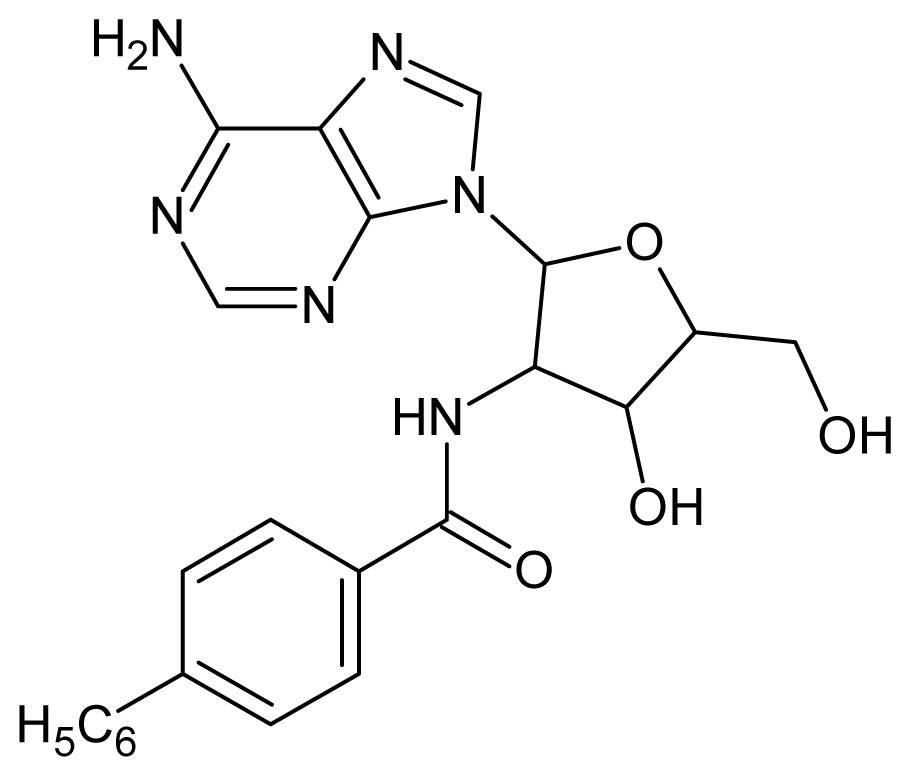	3.60	**32**	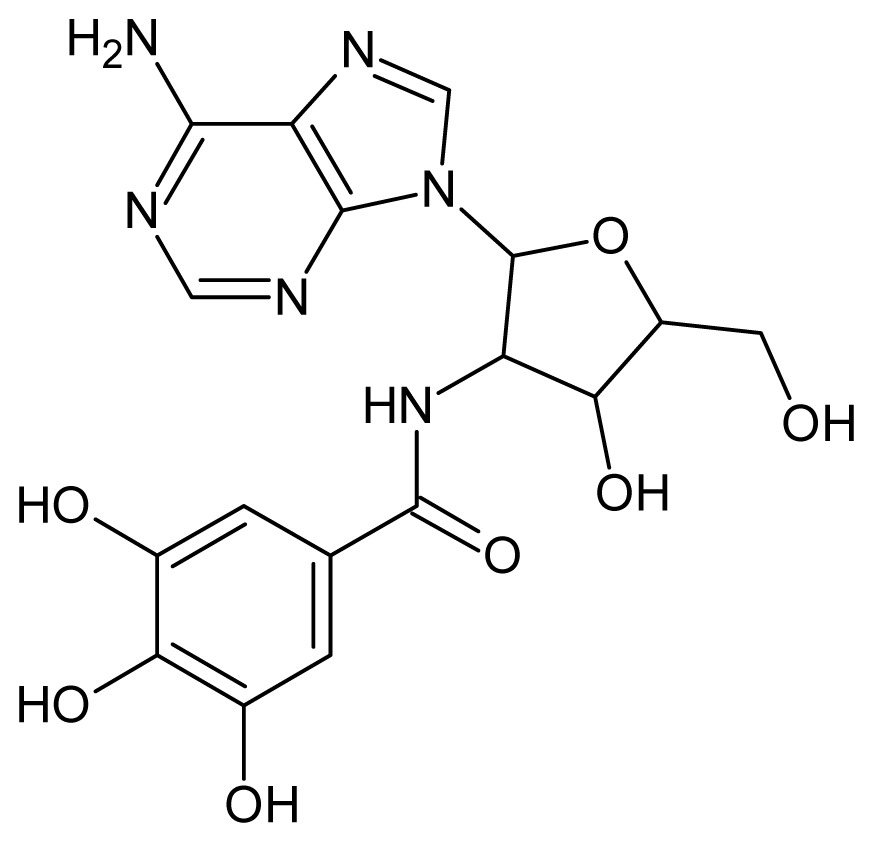	3.60
**33**	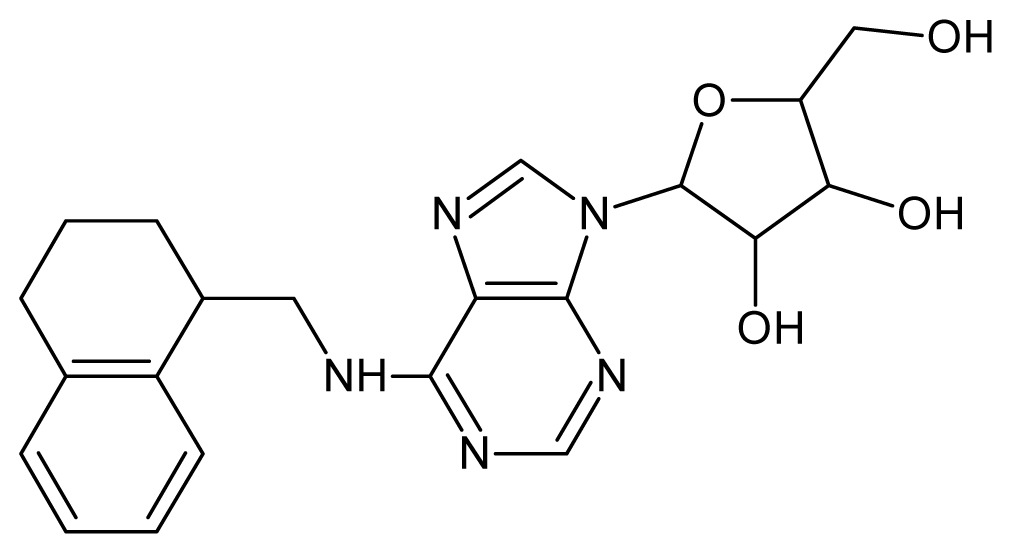	3.44	**34**	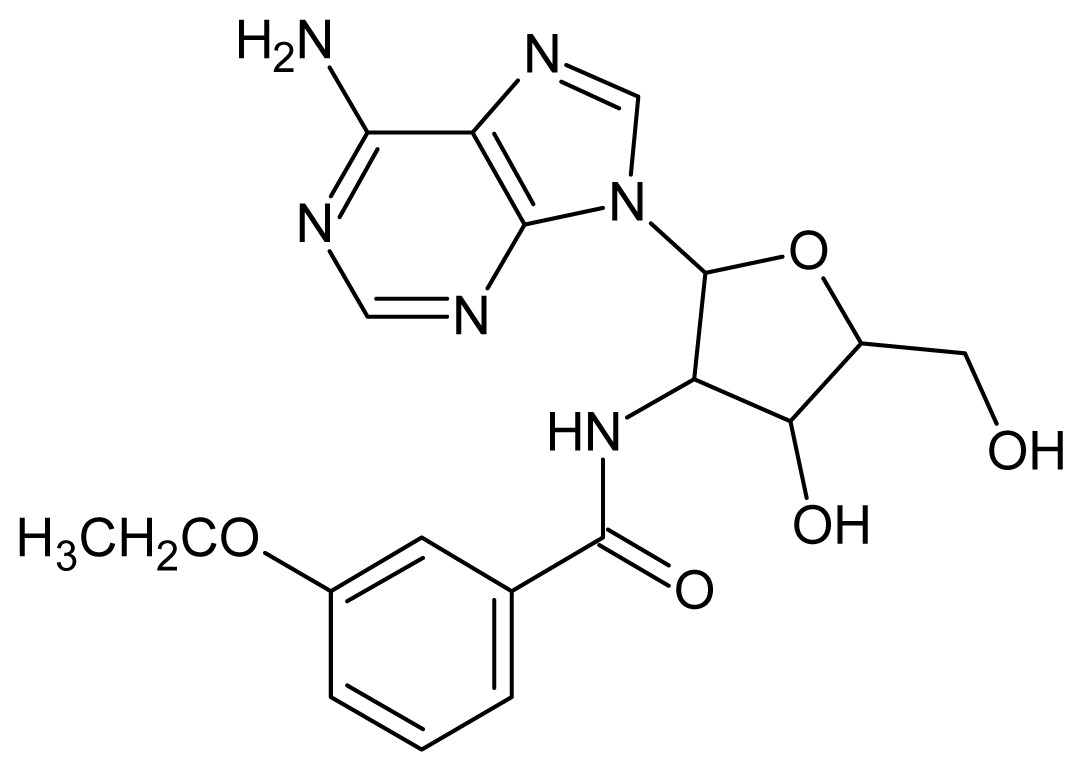	3.40
**35**	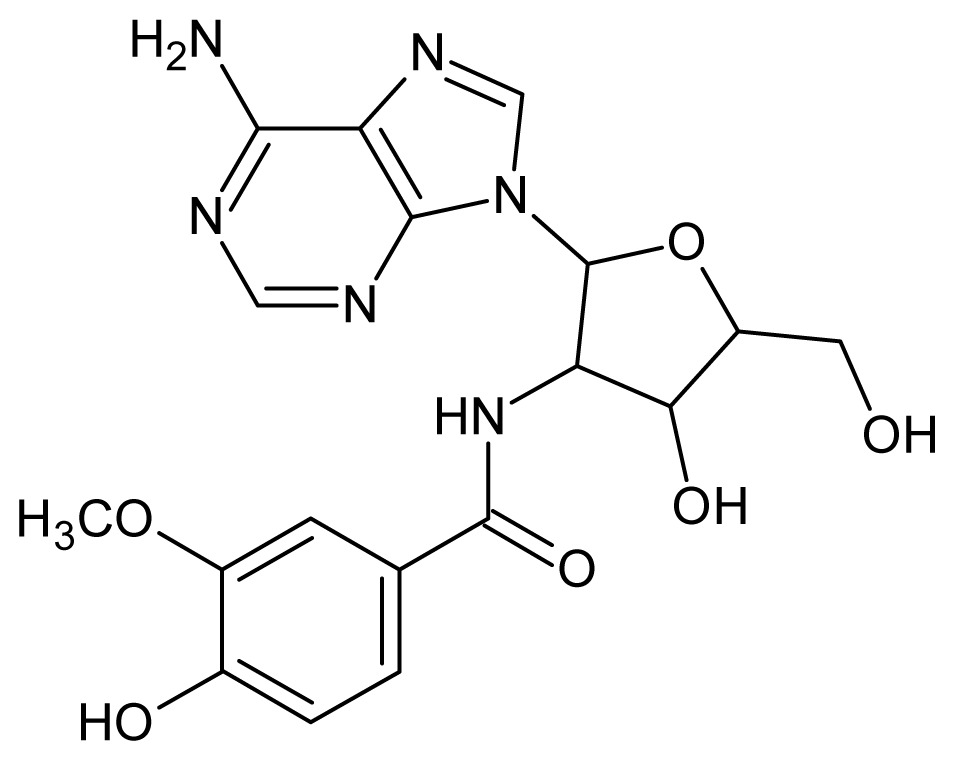	3.40	**36**	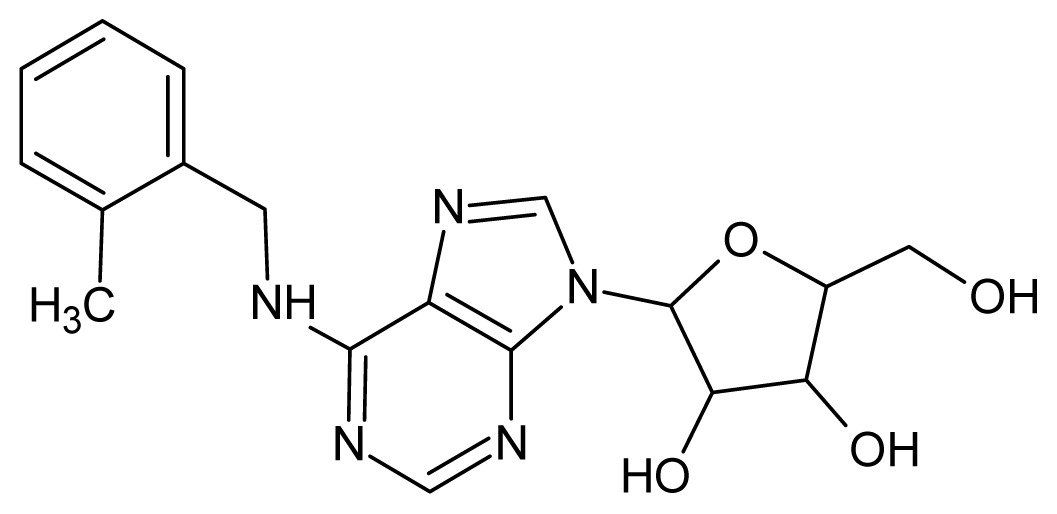	3.30
**37**	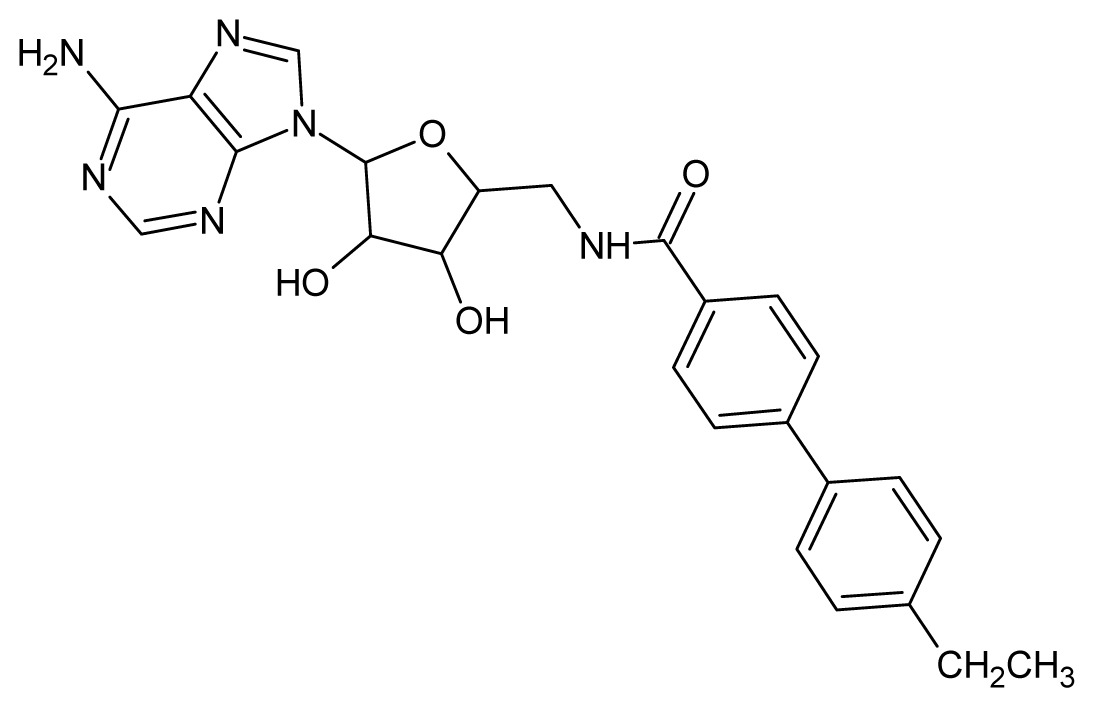	3.30	**38**	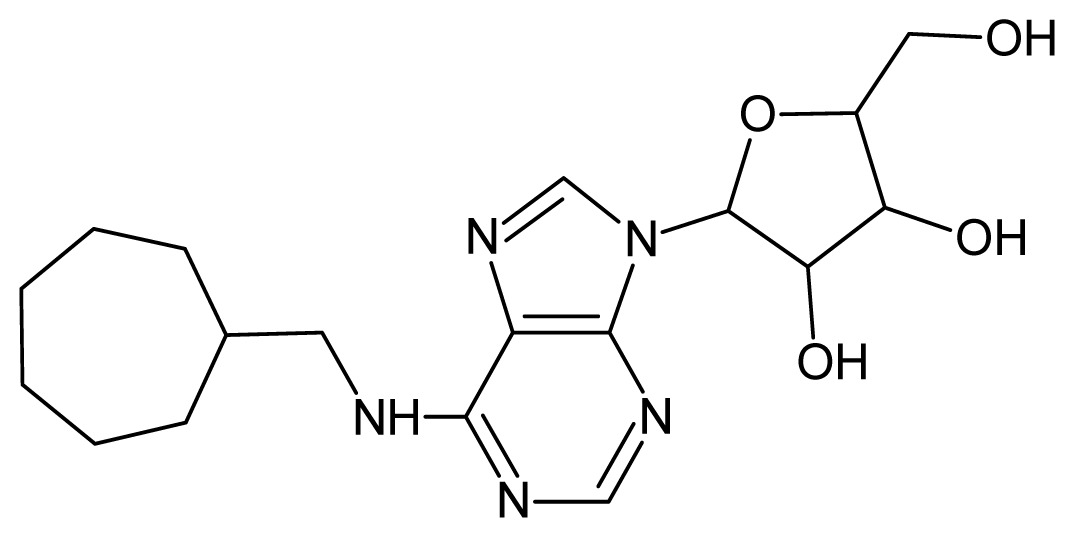	3.15
**39**	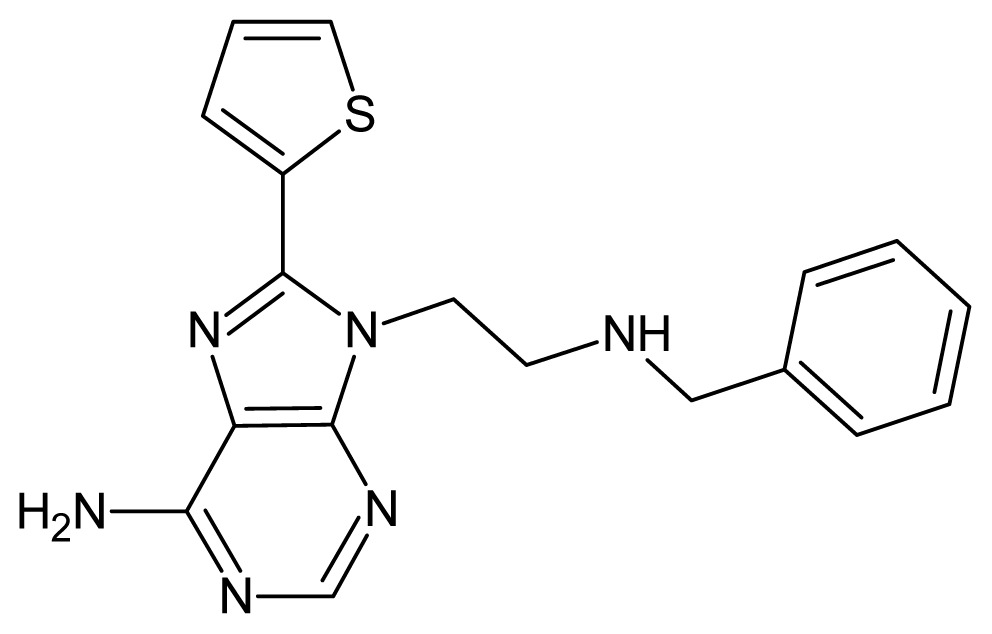	3.15	**40**	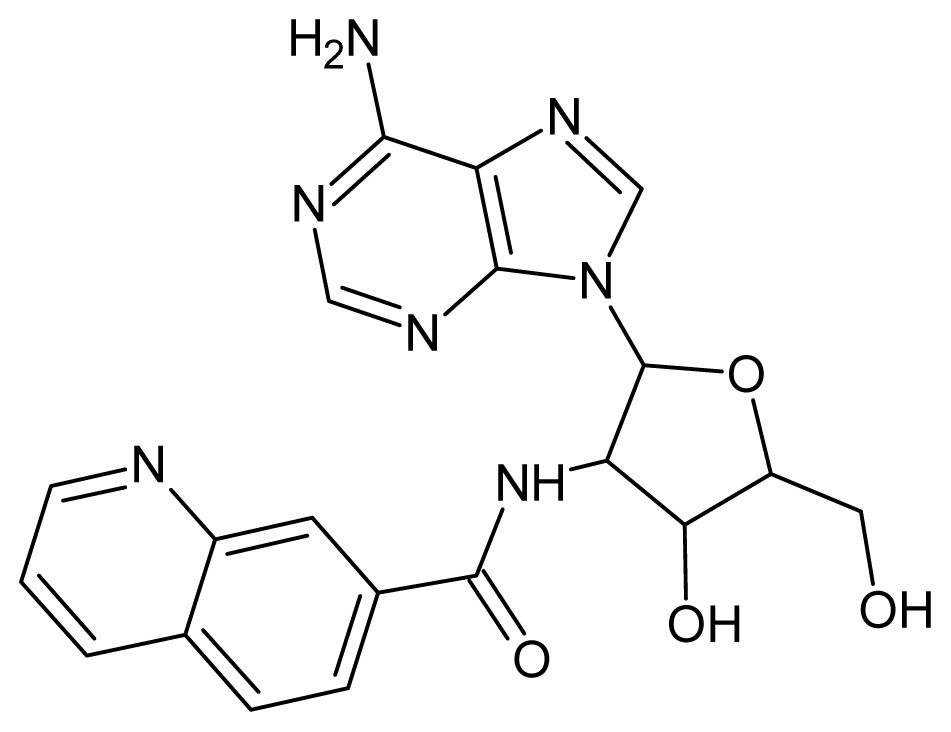	3.15
**41**	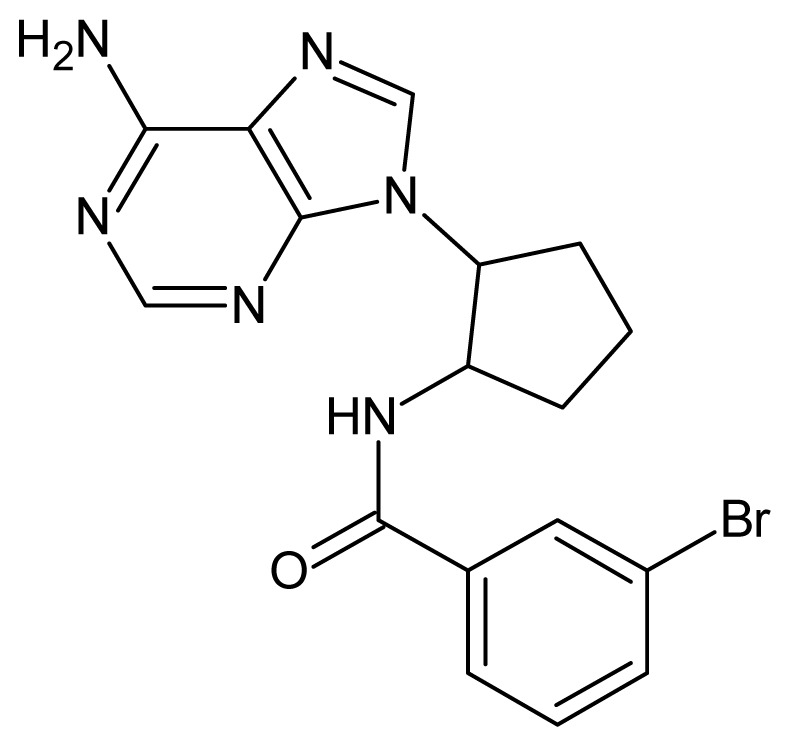	3.12	**42**	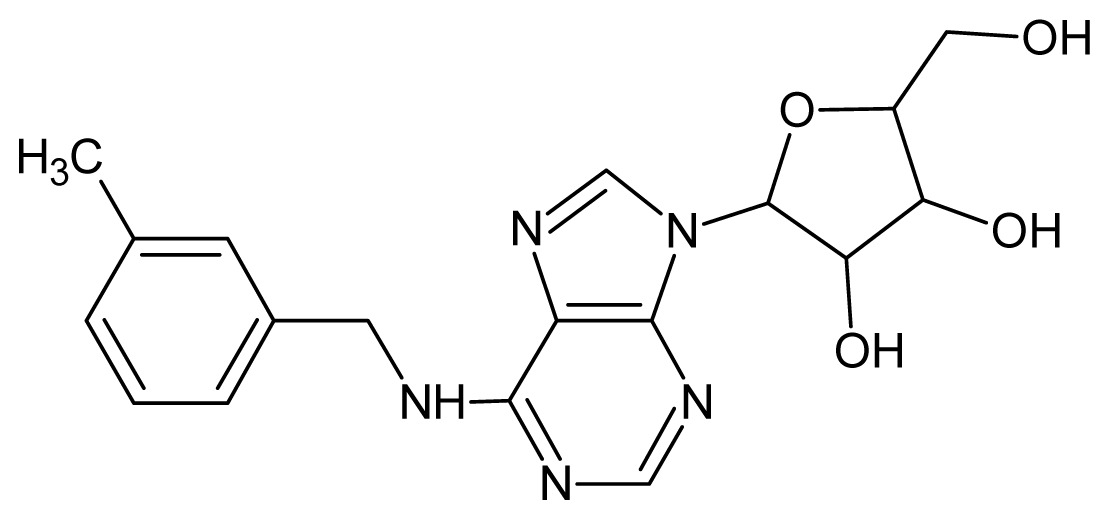	2.80
**43**	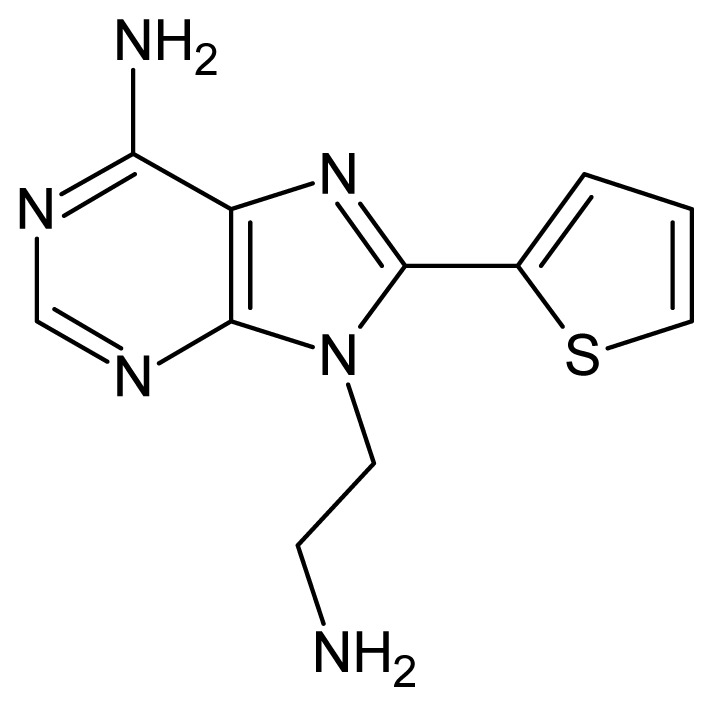	2.74	**44**	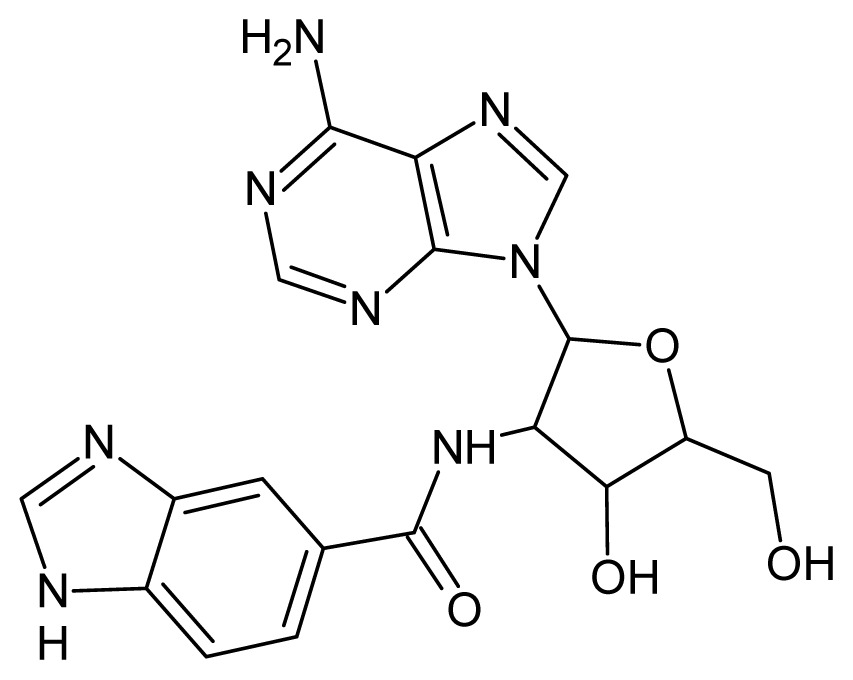	2.52
**45**	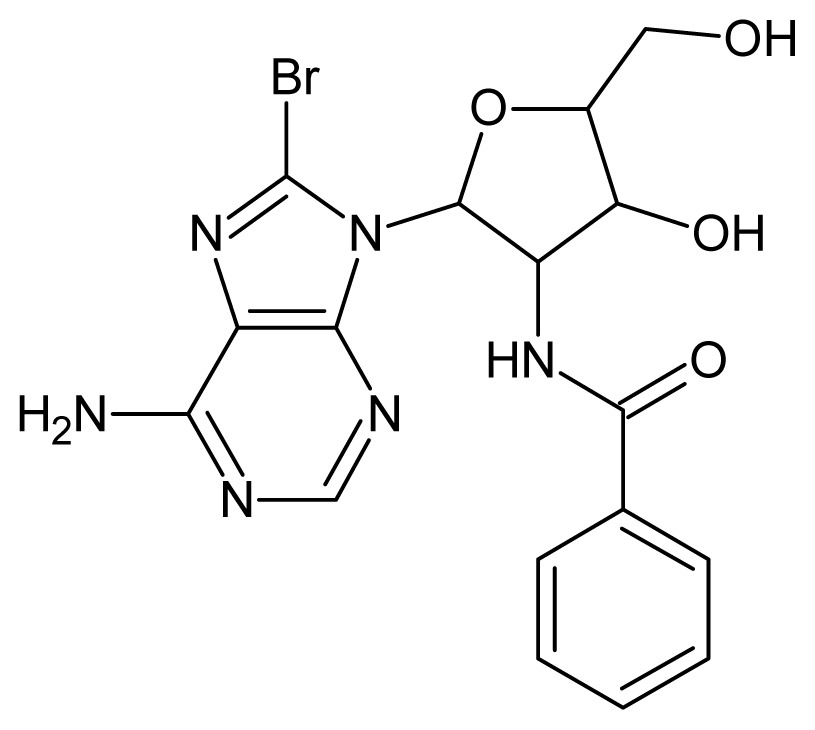	2.52	**46**	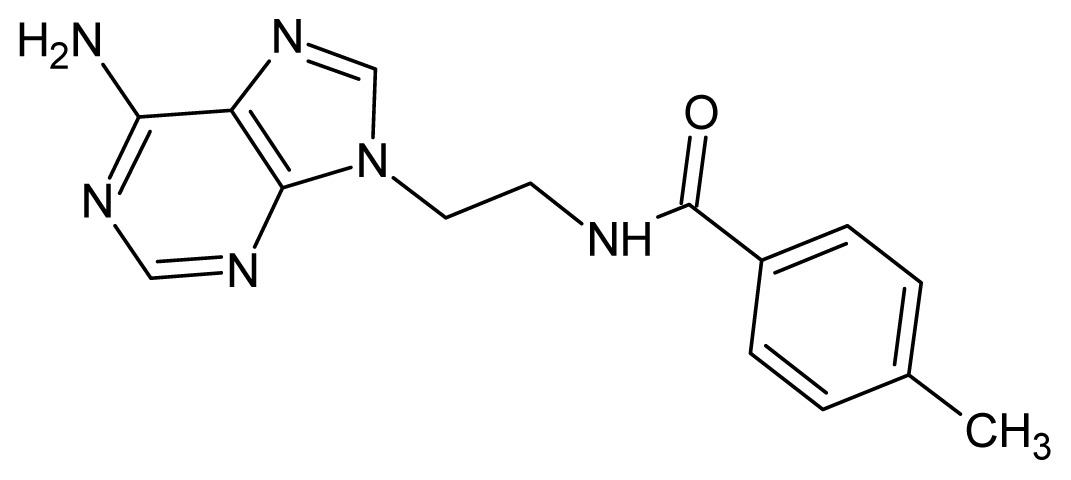	2.48
**47**	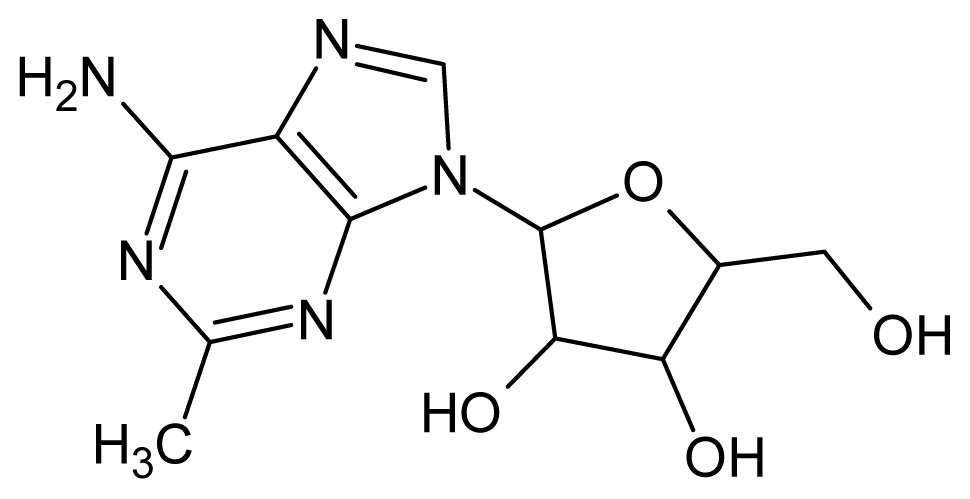	2.40	**48**	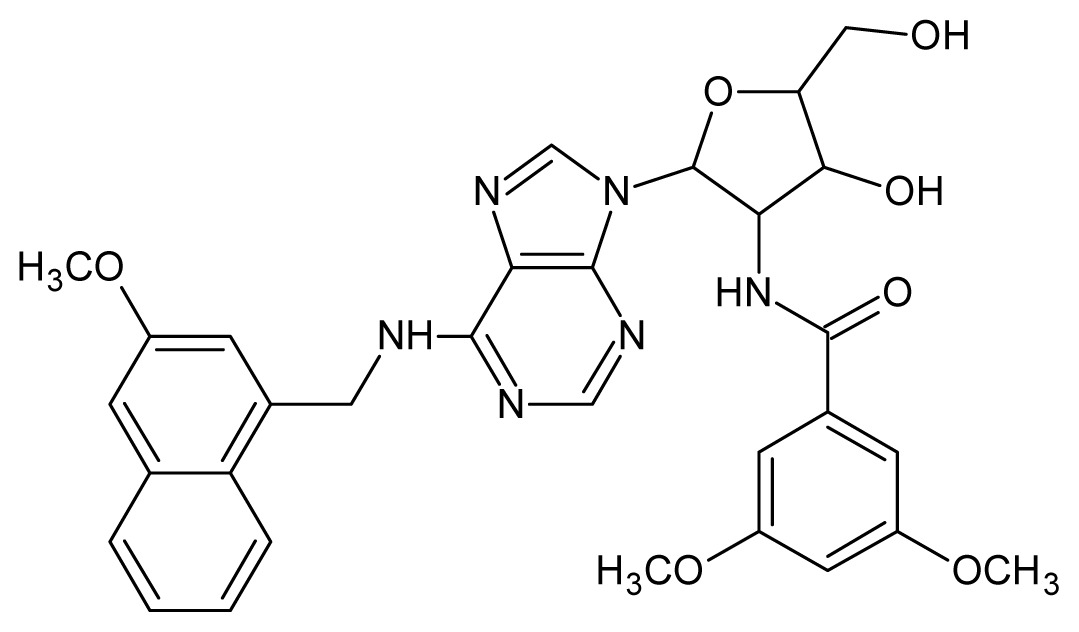	2.40
**49**	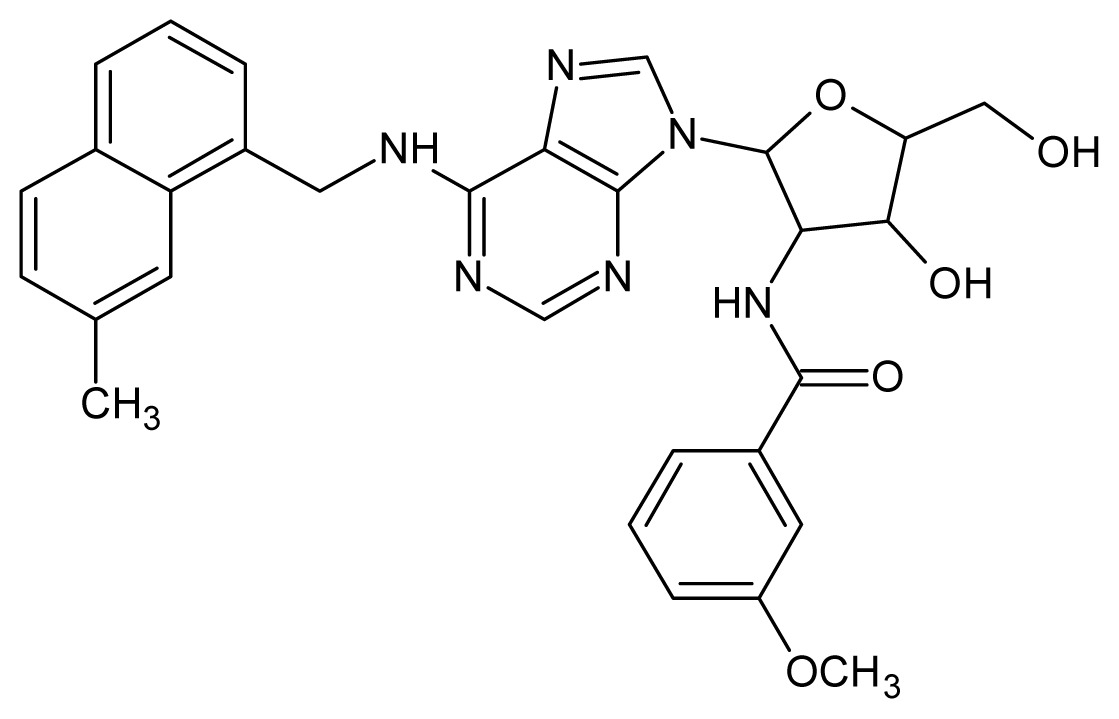	2.22			
**Test Set Compounds**					
**Cpd**	**Structure**	**pIC_50_**	**Cpd**	**Structure**	**pIC_50_**
**50**	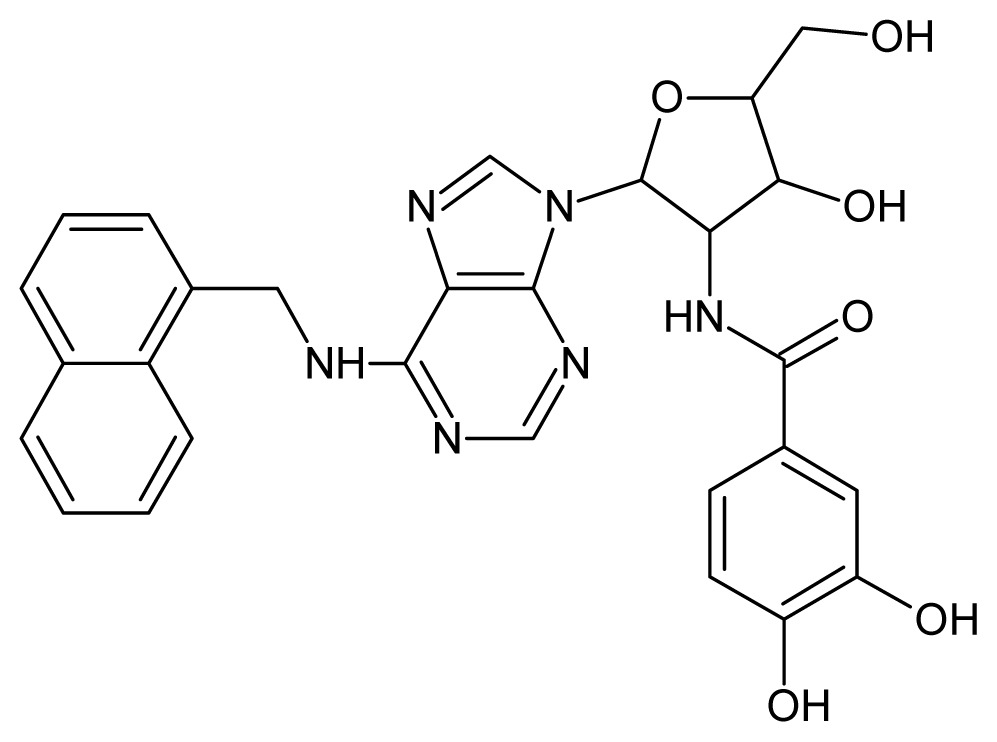	5.70	**51**	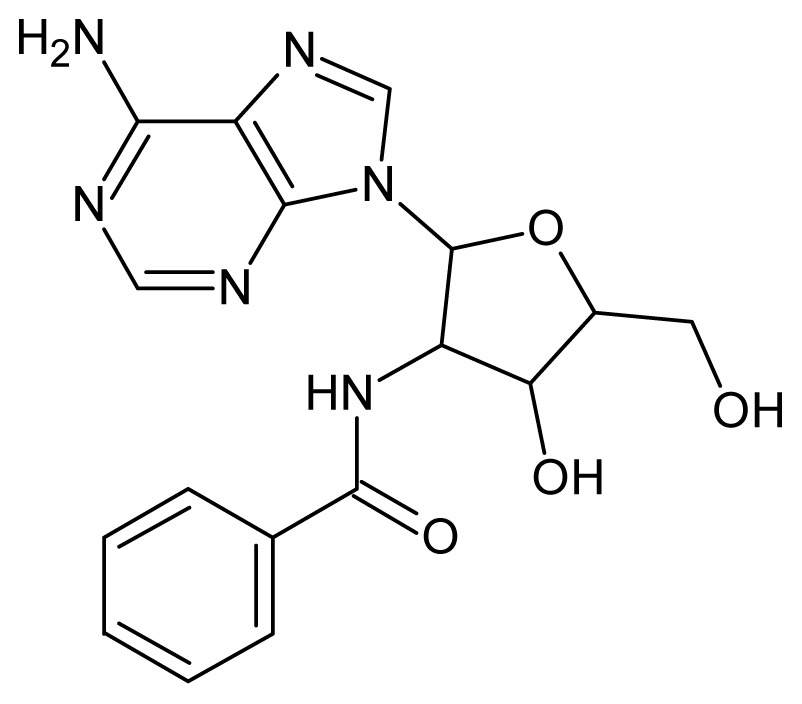	5.40
**52**	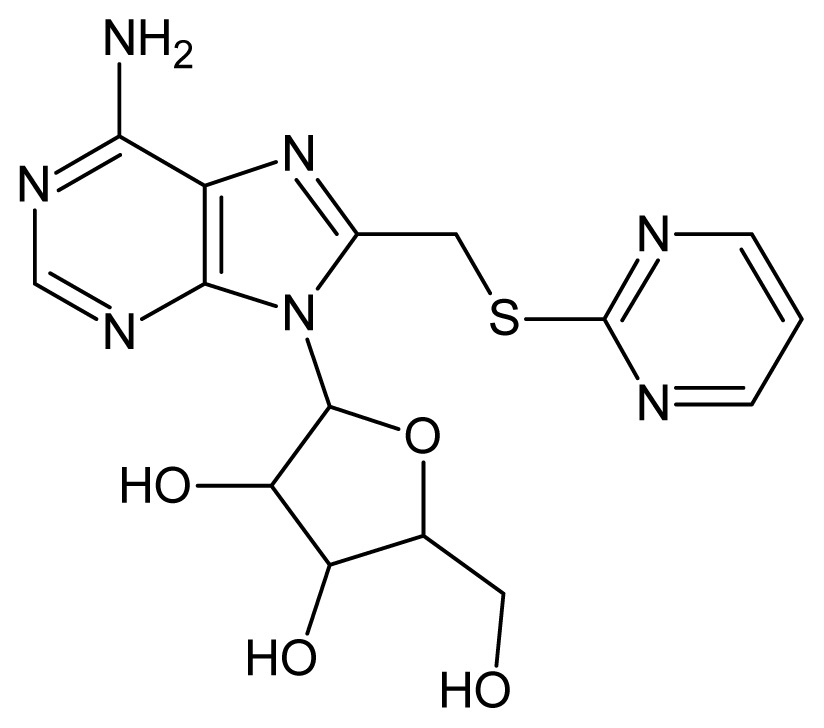	5.30	**53**	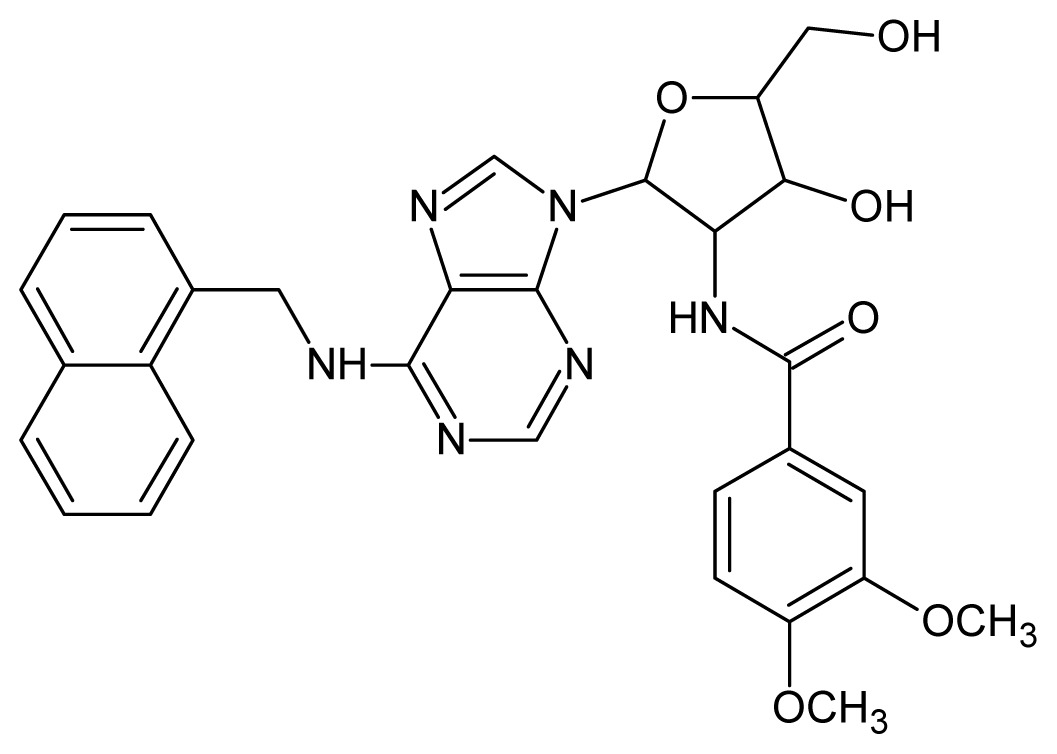	5.00
**54**	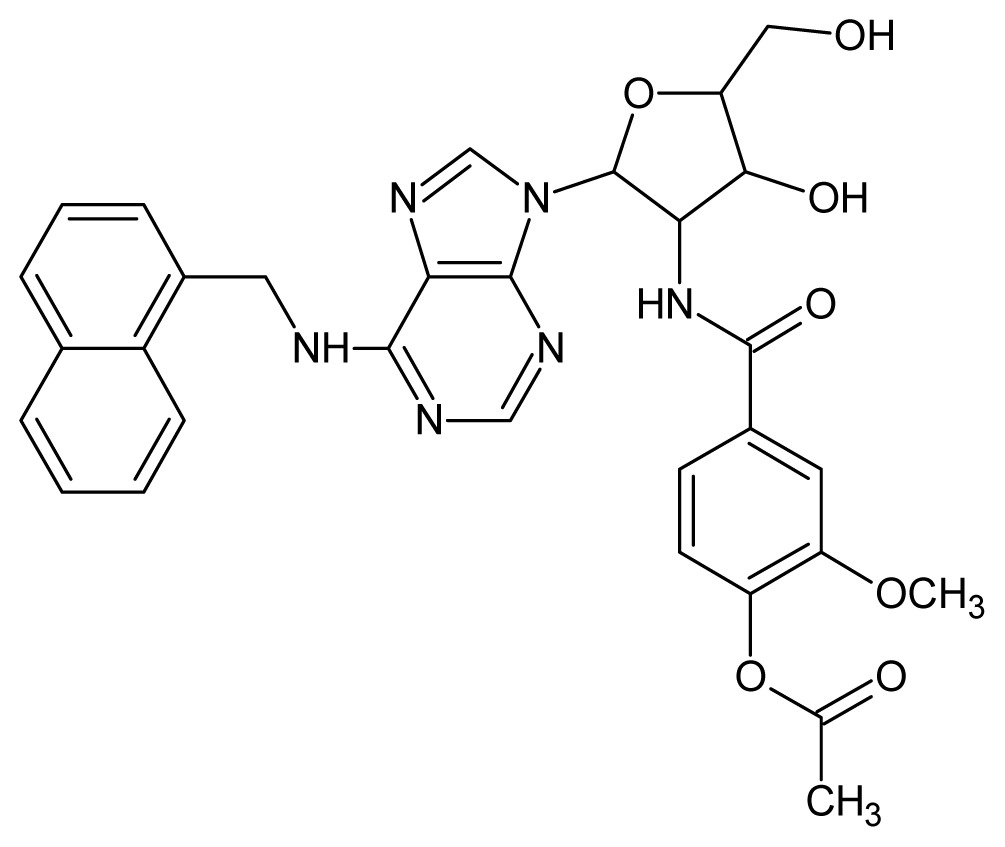	4.74	**55**	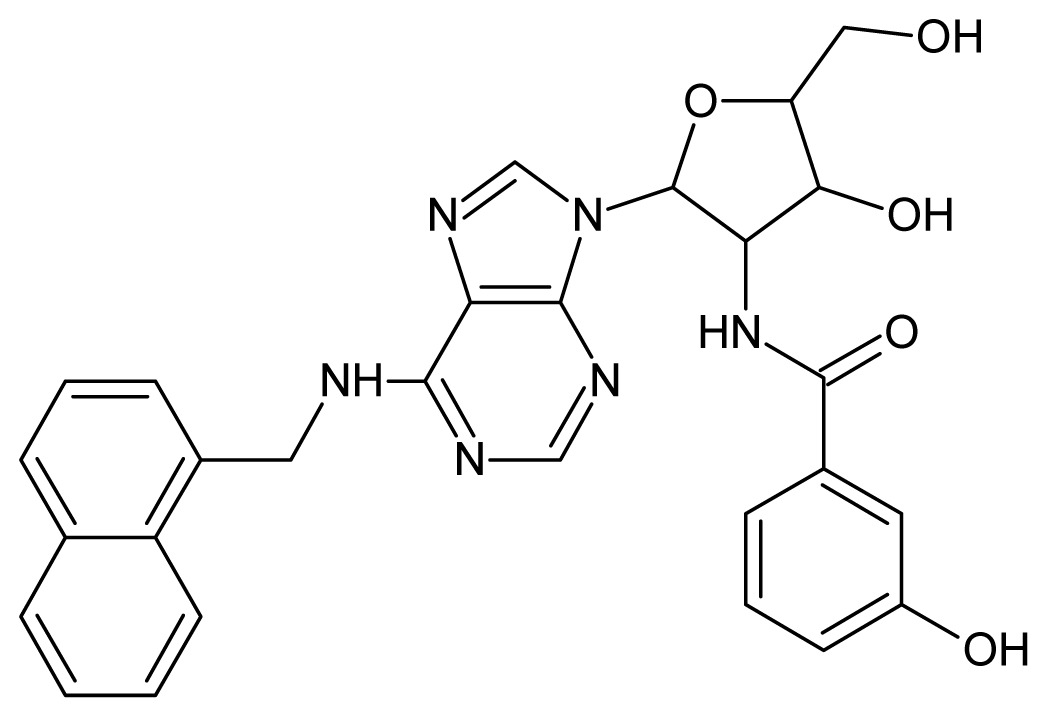	4.60
**Training set compounds**					
**Cpd**	**Structure**	**pIC_50_**	**Cpd**	**Structure**	**pIC_50_**
**56**	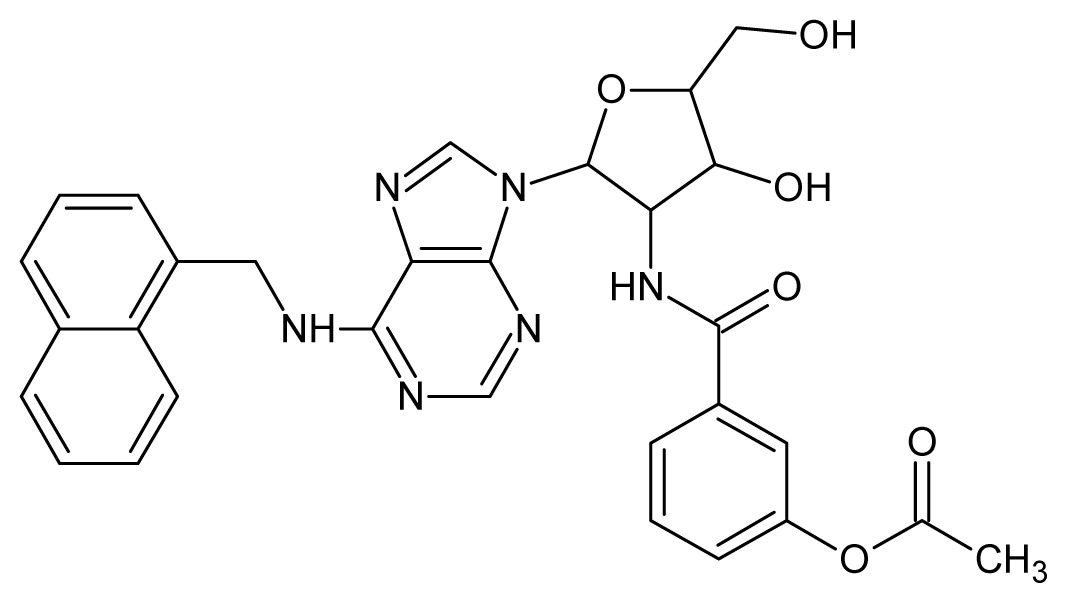	4.30	**57**	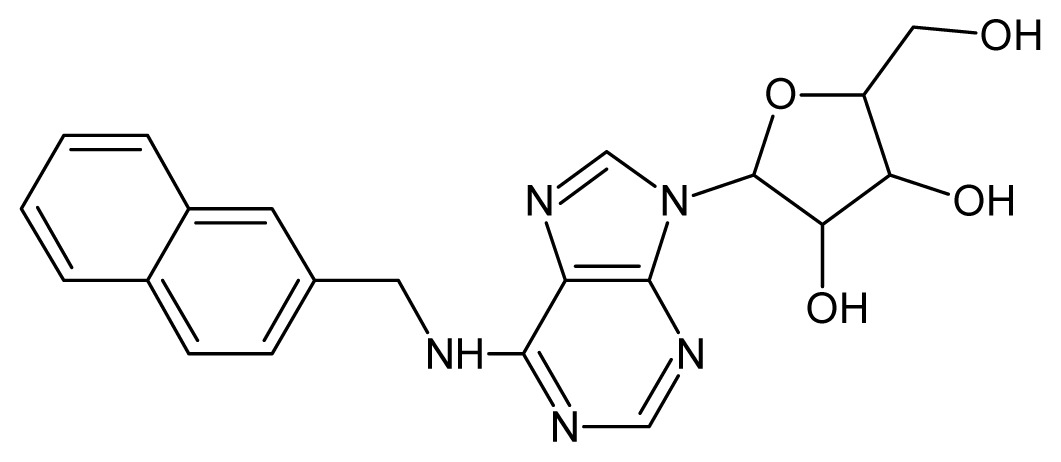	4.00
**58**	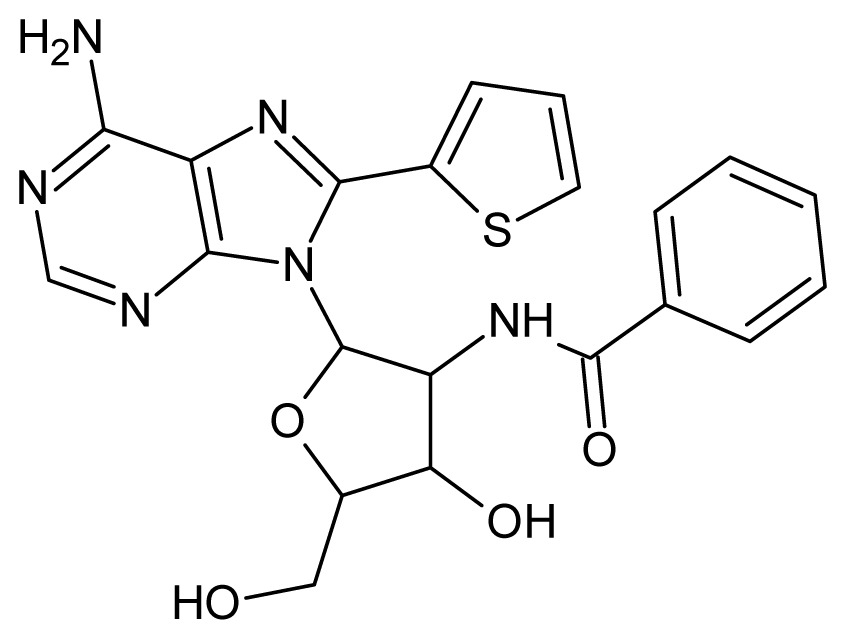	3.82	**59**	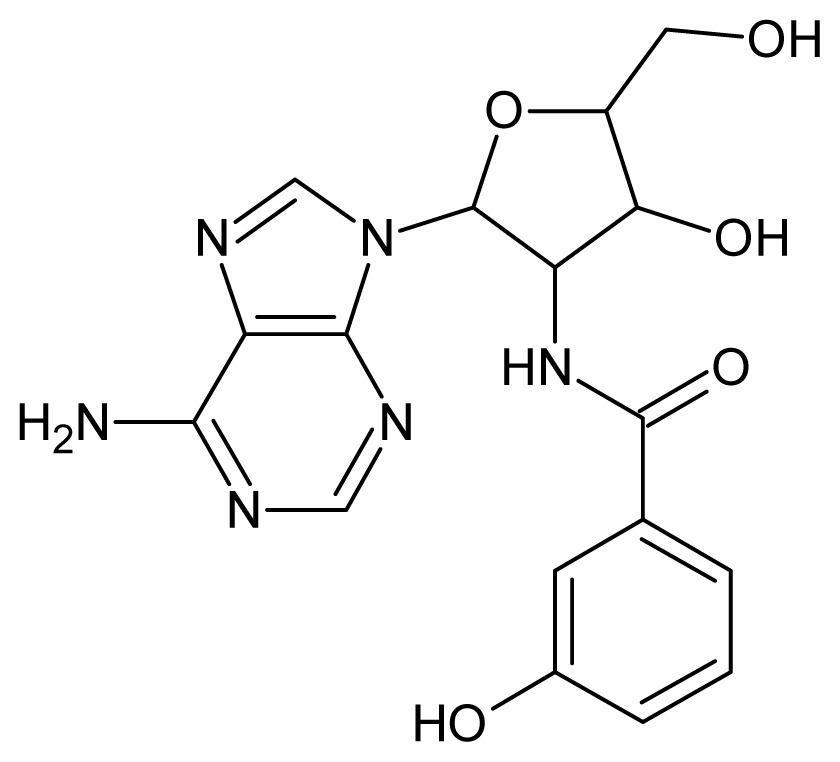	3.52
**60**	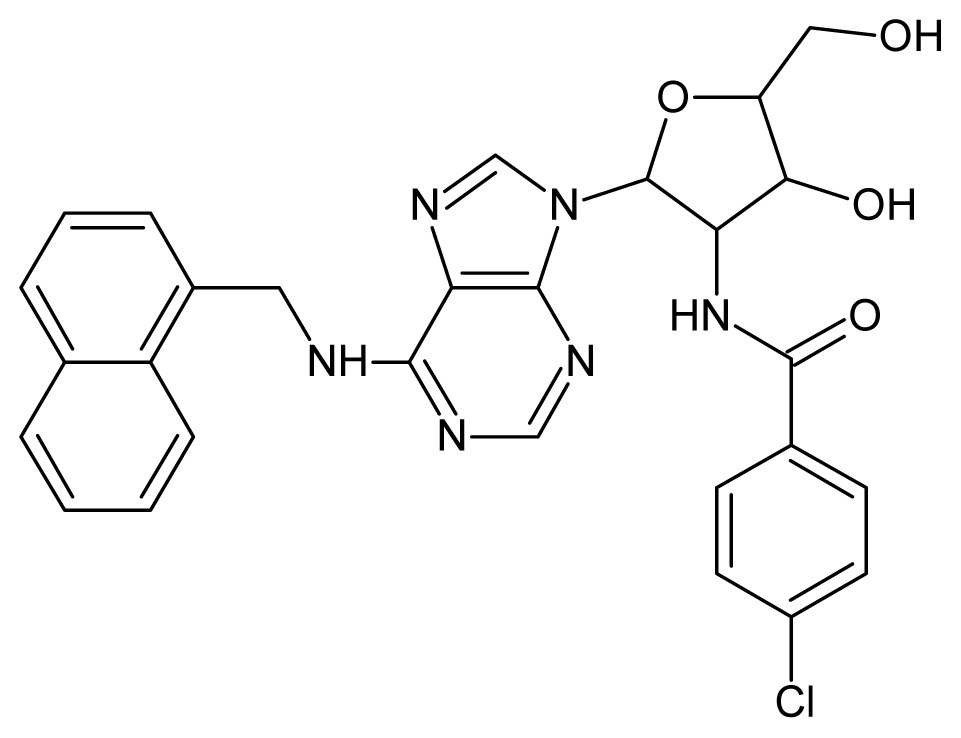	3.30	**61**	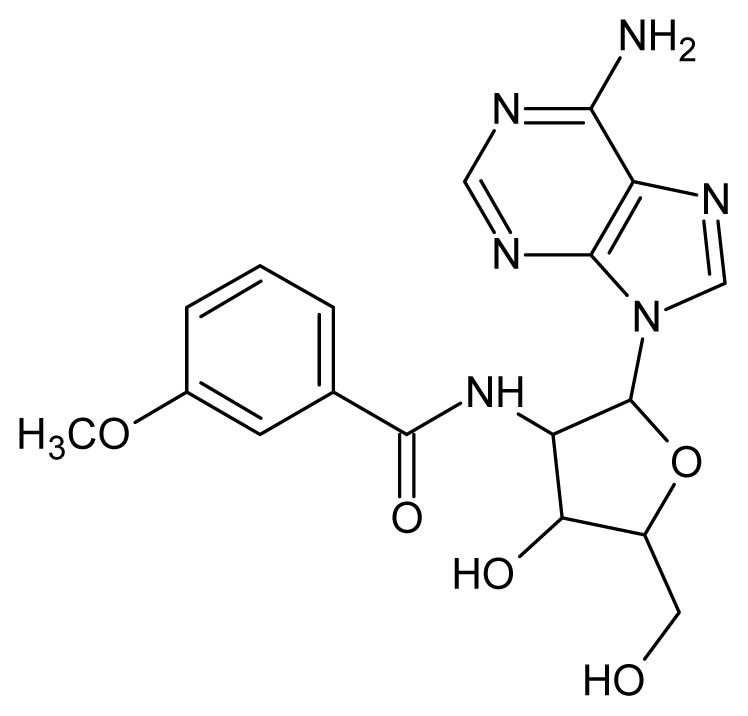	3.22
**62**	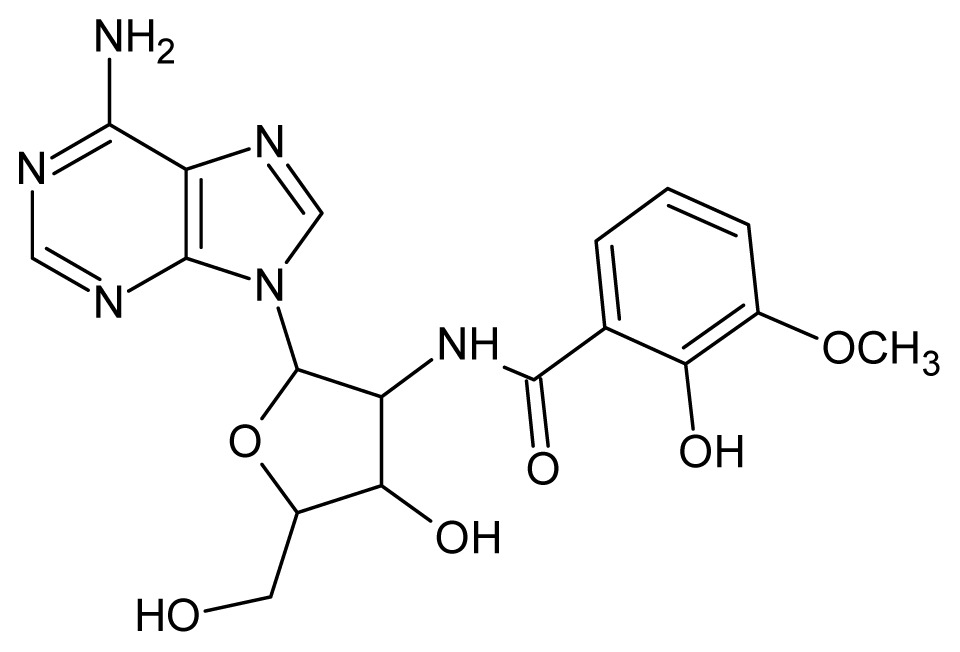	3.07	**63**	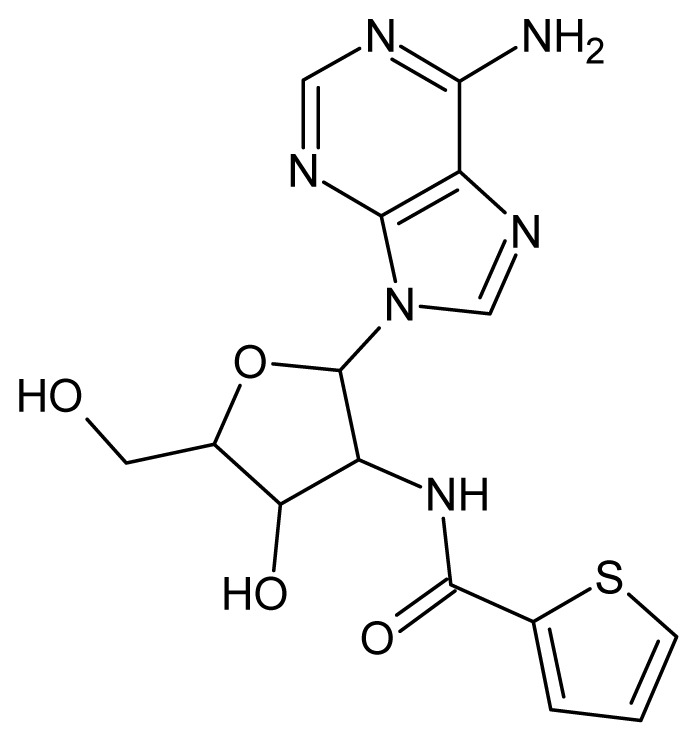	2.62
