# Supplementary material for: Pattern Recognition Techniques Applied to the Study of Leishmanial Glyceraldehyde-3-Phosphate Dehydrogenase Inhibition
Source: Int J Mol Sci. 2014 Feb 21;15(2):3186–203. doi: 10.3390/ijms15023186 (PMC3958905; doi:10.3390/ijms15023186)
Supplement: Supplementary file 1 [file ijms-15-03186-s001.pdf]

Supplementary Information

Table S1. Calculated values of the selected variables for training and test sets.

Compound	E_{LUMO} (a.u.)	QR^2	QR^4	Volume (Å^3)	Polarizability (Å^3)
			Training set		
1	−0.05	−0.58	−0.47	1475.95	57.74
2	−0.05	−0.50	−0.52	1427.44	57.74
3	−0.04	−0.52	−0.52	1436.85	58.38
4	−0.04	−0.55	−0.45	1421.79	57.74
5	0.20	−0.70	−0.53	1388.53	55.36
6	−0.04	−0.59	−0.51	1362.46	54.07
7	−0.04	−0.51	−0.45	1421.40	57.74
8	−0.04	−0.42	−0.50	1365.96	52.76
9	−0.04	−0.55	−0.46	1366.94	55.91
10	−0.06	−0.54	−0.44	1354.56	54.73
11	−0.07	−0.71	−0.27	1474.13	62.53
12	−0.04	−0.54	−0.57	1382.26	55.91
13	−0.07	−0.53	−0.47	1398.68	56.66
14	−0.04	−0.53	−0.51	1298.70	51.56
15	−0.04	−0.52	−0.52	1342.81	53.40
16	−0.04	−0.35	−0.43	1301.86	51.56
17	−0.04	−0.57	−0.56	1404.80	53.40
18	−0.04	−0.51	−0.46	1321.06	52.76
19	−0.08	−0.38	−0.20	1453.44	62.86
20	−0.05	−0.55	−0.31	1413.86	57.82
21	−0.07	−0.53	−0.47	1398.07	56.66
22	0.17	−0.68	−0.58	1411.68	57.29
23	−0.06	−0.51	−0.60	1465.41	59.13
24	0.21	−0.74	−0.52	1416.95	55.91
25	−0.05	−0.50	−0.53	1427.55	57.74
26	−0.04	−0.53	−0.52	1327.97	52.76
27	−0.04	−0.56	−0.48	1340.68	53.44
28	−0.04	−0.51	−0.47	1429.05	58.46
29	−0.05	−0.46	−0.51	1382.38	57.83
30	−0.05	−0.86	−0.46	1185.77	46.62
31	−0.07	−0.91	−0.42	1201.97	48.45
32	−0.04	−0.86	−0.57	1042.74	38.87
33	−0.03	−0.63	−0.37	1079.81	40.89
34	−0.04	−0.97	−0.20	944.67	39.41

Table S1. *Cont.*

Compound	E_{LUMO} (a.u.)	QR^2	QR^4	Volume (Å^3)	Polarizability (Å^3)
		Training set			
35	−0.04	−0.87	−0.44	1069.62	41.26
36	−0.07	−0.88	−0.41	1067.54	42.43
37	−0.04	−0.86	−0.56	1038.91	40.07
38	−0.02	−0.97	−0.52	984.52	37.67
39	−0.03	−0.81	−0.35	989.99	36.25
40	−0.04	−0.57	−0.32	985.22	36.16
41	−0.04	−0.86	−0.51	1303.37	50.29
42	−0.06	−0.94	−0.41	733.28	27.92
43	−0.03	−0.68	−0.33	1022.22	36.73
44	−0.04	−0.90	−0.17	1063.16	40.30
45	−0.05	−0.94	−0.43	1008.78	39.58
46	−0.04	−0.87	−0.47	972.85	36.96
47	−0.04	−0.90	−0.17	873.86	32.15
48	−0.06	−0.88	−0.54	950.52	35.90
49	−0.02	−0.83	−0.27	771.80	26.50
		Test set			
50	−0.04	−0.68	−0.49	1549.31	62.05
51	−0.07	−0.48	−0.52	1516.73	59.58
52	−0.04	−0.49	−0.49	1493.03	58.94
53	−0.05	−0.45	−0.52	1539.07	61.50
54	−0.05	−0.47	−0.52	1394.89	55.91
55	0.21	−0.76	−0.53	1394.45	55.27
56	0.21	−0.66	−0.60	1467.98	59.03
57	−0.05	−0.48	−0.54	1403.44	56.56
58	−0.04	−0.53	−0.33	1082.42	41.88
59	−0.04	−0.87	−0.48	1053.74	39.43
60	−0.06	−0.90	−0.50	1134.25	46.27
61	−0.03	−0.87	−0.56	1050.11	40.07
62	−0.03	−0.88	−0.47	997.36	37.59
63	−0.06	−0.87	−0.59	938.91	36.48

© 2014 by the authors; licensee MDPI, Basel, Switzerland. This article is an open access article distributed under the terms and conditions of the Creative Commons Attribution license (http://creativecommons.org/licenses/by/3.0/).
